# Addressing non-medical health-related social needs through a community-based lifestyle intervention during the COVID-19 pandemic: The Black Impact program

**DOI:** 10.1371/journal.pone.0282103

**Published:** 2023-03-09

**Authors:** Joshua J. Joseph, Darrell M. Gray, Amaris Williams, Songzhu Zhao, Alicia McKoy, James B. Odei, Guy Brock, Dana Lavender, Daniel M. Walker, Saira Nawaz, Carrie Baker, Jenelle Hoseus, Tanikka Price, John Gregory, Timiya S. Nolan

**Affiliations:** 1 The Ohio State University College of Medicine, Columbus, Ohio, United States of America; 2 The Ohio State University James Center for Cancer Health Equity, Columbus, Ohio, United States of America; 3 The Ohio State University College of Public Health, Columbus, Ohio, United States of America; 4 The African American Male Wellness Agency, National Center for Urban Solutions, Columbus, Ohio, United States of America; 5 Healthcare Collaborative of Greater Columbus, Columbus, Ohio, United States of America; 6 The Ohio State University College of Nursing, Columbus, Ohio, United States of America; Medical College of Wisconsin, UNITED STATES

## Abstract

**Background:**

Non-medical health-related social needs (social needs) are major contributors to worse health outcomes and may have an adverse impact on cardiovascular risk factors and cardiovascular disease. The present study evaluated the effect of a closed-loop community-based pathway in reducing social needs among Black men in a lifestyle change program.

**Methods:**

Black men (n = 70) from a large Midwestern city participated in Black Impact, a 24-week community-based team lifestyle change single-arm pilot trial adapted from the Diabetes Prevention Program and American Heart Association’s (AHA) Check, Change, Control Blood Pressure Self-Management Program, which incorporates AHA’s Life’s Simple 7 (LS7) framework. Participants were screened using the Centers for Medicare and Medicaid Services (CMS) Accountable Health Communities Health-Related Social Needs Screening Tool. Participants with affirmative responses were referred to a community hub pathway to address social needs. The primary outcome for this analysis is change in social needs based on the CMS social needs survey at 12 and 24 weeks using mixed effect logistic regressions with random intercepts for each participant. Change in a LS7 score (range 0–14) from baseline to 12 and 24 weeks was evaluated using a linear mixed-effects model stratified by baseline social needs.

**Results:**

Among 70 participants, the mean age of participants was 52 ±10.5 years. The men were sociodemographically diverse, with annual income ranging from <$20,000 (6%) to ≥$75,000 (23%). Forty-three percent had a college degree or higher level of education, 73% had private insurance, and 84% were employed. At baseline 57% of participants had at least one social need. Over 12 and 24 weeks, this was reduced to 37% (OR 0.33, 95%CI: 0.13, 0.85) and 44% (OR 0.50, 95%CI: 0.21, 1.16), respectively. There was no association of baseline social needs status with baseline LS7 score, and LS7 score improved over 12 and 24 weeks among men with and without social needs, with no evidence of a differential effect.

**Conclusions:**

The Black Impact lifestyle change single-arm pilot program showed that a referral to a closed-loop community-based hub reduced social needs in Black men. We found no association of social needs with baseline or change in LS7 scores. Further evaluation of community-based strategies to advance the attainment of LS7 and address social needs among Black men in larger trials is warranted.

## Introduction

Non-medical health-related social needs (social needs) are individual social and economic needs such as housing, food, transportation, and protection from violence [1]. Social needs are major contributors to worse health outcomes [2–4] and are estimated to impact up to 50–60% of health outcomes [5]. There is a strong body of evidence supporting social needs as a critical lever toward the achievement of health equity and the need to expand the healthcare sector’s purview beyond the traditional walls of a healthcare system [6]. Interventions to address social needs have been shown to: 1) improve processes (e.g. identification of social needs, referrals, and enrollment in community resources); and 2) lower cost and improve utilization (e.g. improved preventive care utilization, decreased length of stay and hospital readmissions) [7–10]. Evidence of health improvements after addressing social needs is mixed, with some studies showing an improvement in blood pressure, lipids, and fruit/vegetable consumption, while other studies did not show improvement in glycemic measures [7–10]. Emerging data shows that higher intervention dosage (number of contacts between the navigator and patient/participant) may be related to greater success of resource connections, with in-person contact being associated with the highest likelihood of success [11].

In the United States, the prevalence of social needs is higher among racial/ethnic minority groups, which impacts heart healthy behaviors. Black, Latino, and Filipino adults in the Kaiser Permanente Northern California integrated primary and specialty health care network were more likely than Whites to be in a lower income category and worry about their financial situation [12]. Cost-related reduced medication use was higher among Black individuals, and cost-related reduced fruit/vegetable consumption was higher among Black and Latino populations [12]. Racial/ethnic disparities in income were observed within similar levels of education [12]. In Black adults in the Jackson Heart Study, lower individual income, neighborhood socioeconomic status, and education were all significantly associated with lower American Heart Association (AHA) Life’s Simple 7 (LS7) scores (LS7 metrics include physical activity, diet, cholesterol, blood pressure, body mass index (BMI), smoking, and glycemia) [13]. In community-dwelling Black men participating in African American Male Wellness Walks, lower annual income (<$20,000 vs. ≥$75,000) and Medicare or no insurance vs. private insurance were associated with worse AHA cardiovascular health [14]. Unfortunately, interventions addressing social needs and cardiovascular risk factors have been limited in all populations, including racial/ethnic minority groups. This gap is troubling because in the United States, Black men have lower attainment of ≥ 5 AHA LS7 metrics compared to women and non-Hispanic White (White) populations [15]. Higher AHA LS7 scores are associated with lower risk of cardiovascular disease, type 2 diabetes (diabetes), cancer, and mortality among all races/ethnicities [16–19]. Thus, addressing social needs as one avenue to improve cardiovascular risk factors is critical given the widening racial disparities in preventable deaths from heart disease and stroke [20,21].

Our research group maintains a community-engaged and community-based focus founded in academic-community-government partnerships to advance health [22–24]. In a systematic review, we found no evidence of previous community-based participatory research (CBPR) approaches focused on LS7 in Black men [22]. Thus, using a CBPR approach with our community partner, The National African American Male Wellness Agency (AAMWA) and community members, we co-designed Black Impact, a 24-week CBPR study which improved LS7 attainment in Black men residing in a large Midwestern city [25]. Black Impact had 3 main components: 1) 24-week physical activity, nutrition and education intervention in Black men [25]; 2) Navigating participants without a primary care provider to establish care with a provider and improve patient activation; and 3) Addressing social needs that present barriers to wellness. The current report evaluates the baseline social needs screening, referral and outcomes and the impact on cardiovascular health scores. The study team hypothesized that: 1) participants’ social needs would improve over the course of the intervention; 2) participants with social needs would have worse cardiovascular health at baseline; and 3) baseline social needs would lower the magnitude of improvement in cardiovascular health scores.

## Materials and methods

### Study design and recruitment

**Fig 1:**
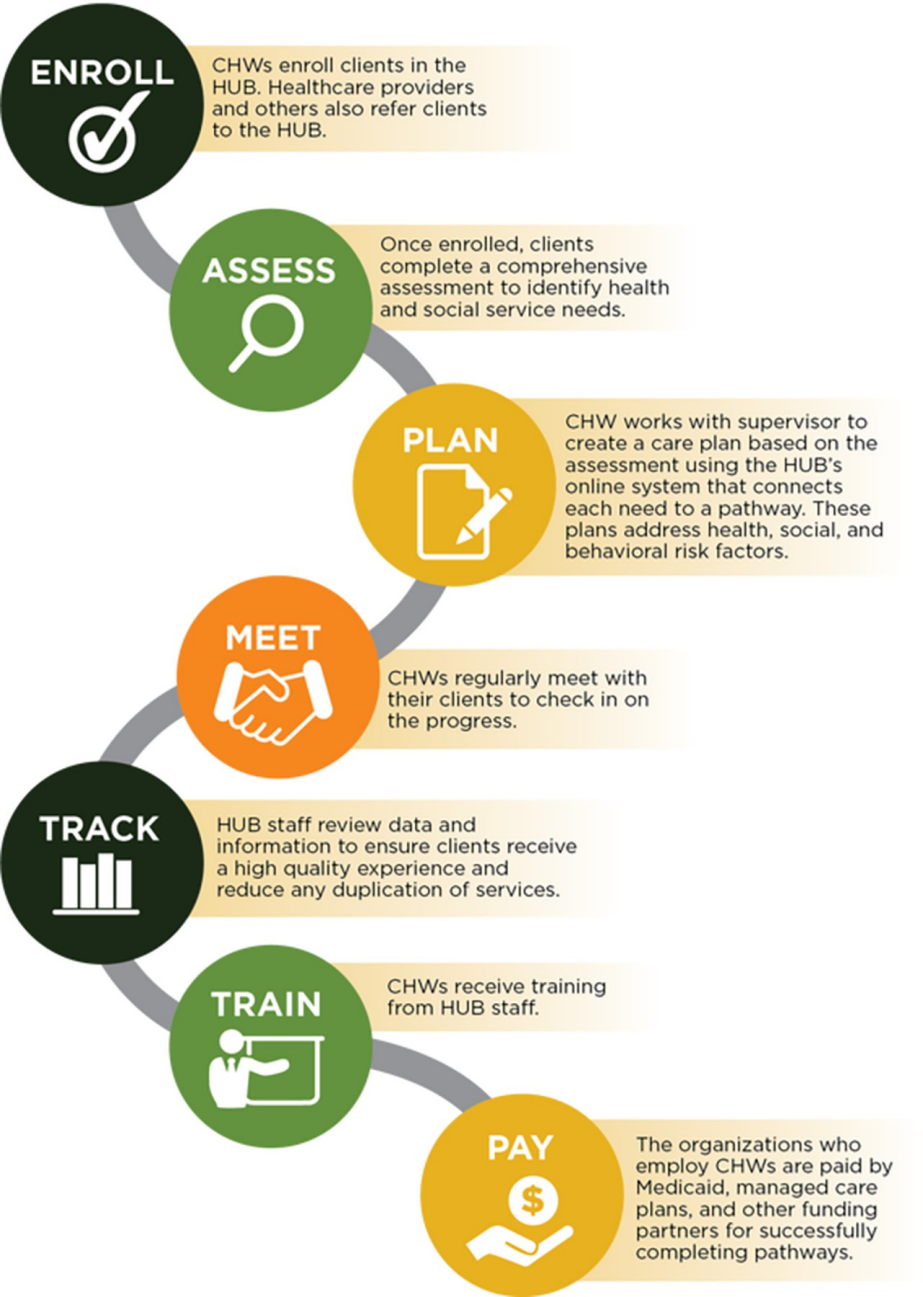
Consort 2010 flow diagram.

As has been described previously and is shown in Fig 1 [25], we enrolled Black men from the annual AAMWA walk/health fair with poor or average cardiovascular health (< 4 LS7 metrics in the ideal range). The inclusion criteria included: 1) Black men (self-report); 2) adult age 18 years or older; 3) poor or average cardiovascular health (< 4 LS7 metrics in the ideal range); English speaking; 5) lives in Metropolitan Columbus, Ohio area; 6) no healthcare provider-imposed limitations on physical activity; and 7) participant has no contraindications for a group setting. In February 2020, 100 Black men were enrolled in the pilot study and divided into 6 geographic-based teams by the study team [26]. The sample size was based on the number of participants needed to determine effect sizes for the primary outcome (50–100 participants) [26]. Due to COVID-19, the study was paused prior to initiation. In July 2020, the study began with 74 participants with programming through December 2020 [25]. The Black Impact programming phase was implemented over 24 weeks from July 2020 to December 2020. Twelve- and 24-week biometric health screenings occurred at study sites, and survey data were collected electronically via Research Electronic Data Capture (REDCap). The study was reviewed and approved by The Ohio State University Biomedical Sciences Institutional Review Board (Study ID: 2019H0302) and was retrospectively registered on ClinicalTrials.gov Identifier: NCT04787978 on March 9, 2021. The principal investigators were unaware of the necessity for clinical trial registration of pilot single-arm clinical trials at study commencement and confirm that future trials will be prospectively registered. All participants provided written informed consent.

### Intervention

The 24-week community-based lifestyle intervention focused on health education, physical activity and addressing social needs through screening and service coordination aimed to improve cardiovascular health among Black men. This single-arm pilot study was adapted from the Diabetes Prevention Program [27] and AHA Check, Change, Control programs, applying evidence-based strategies and stakeholder feedback [28]. Thus, participants were not randomized, and all received the entire intervention using a single-arm trial design [25,26]. Each participant was assigned to a health coach and grouped into six teams of 8–25 participants based on participant proximity to a central meeting location (e.g., Columbus Recreation and Parks recreation center). The Black Impact physical activity, nutrition, and education intervention has been described previously [25]. The Black Impact intervention was grounded in the social cognitive theory at the individual level, and used a multi-level framework consistent with the socioecological model (individual, interpersonal, organizational, community and policy). Our research team used the PETAL framework for CBPR: 1) prioritize health equity; 2) engage the community; 3) target health disparities; 4) act on the data; and 5) learn and improve [22–24,29]. As part of the community engagement and co-designing of the intervention, addressing social needs was determined to be a key component, consistent with the work of Kangovi and others [1].

**Fig 2:**
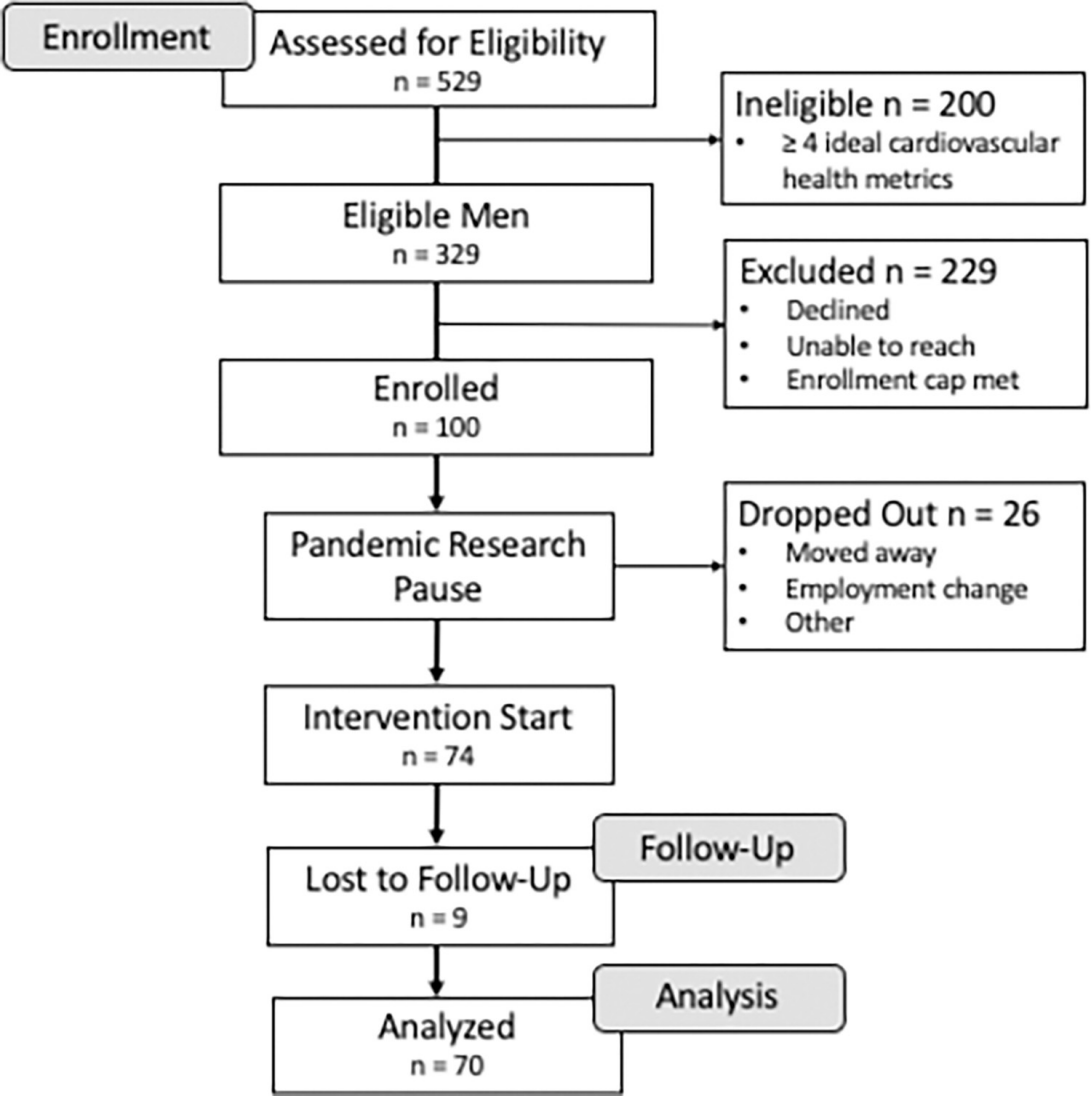
Flow diagram for participants engaged in central ohio pathways hub.

At baseline, participants were screened for social needs using The Center for Medicare and Medicaid (CMS) Accountable Health Communities Health-Related Social Needs Screening Tool by the study team. The screening tool includes 26 questions addressing living situation, food security, transportation, utilities, safety, financial strain, employment, family and community support, education, physical activity, substance abuse, mental health, and disabilities [30,31]. Participants who screened positive for any social needs were referred to the Healthcare Collaborative of Greater Columbus Central (HCGC) Ohio Pathways Hub (Hub) using a secure web interface by Black Impact study staff. Black Impact participants were paired with a community health worker (CHW) from a care coordination agency (CCA) within the HCGC Hub. There were 13 CCAs employing over 30 CHWs participating in the HCGC Hub. CHWs served as partners, advocates, and coaches for their clients and worked to identify health needs and risks. The CHW contacted the study participants and conducted a comprehensive social needs screening assessment which aligned with the CMS screening tool but went into greater depth. Each risk or need was then translated into a pathway, with the CHW guiding participants through the appropriate care pathways, which were tracked in the Care Coordination Systems (CCS) secure data collection platform. CHWs were required to meet face-to-face with each participant monthly as well as have a second contact (phone, text, or email) per month. CHWs continued assisting participants in completing pathways and mitigating risks until participants’ needs were addressed. In the CCS secure data collection platform, referrals from the Black Impact program were flagged into a program designation and pertinent data was aggregated. HCGC reviewed the participants on an ongoing basis via a Checklist, Pathways, Tools (CPT) report and shared progress with Black Impact study staff. The report articulated all comprehensive risk assessments, any care pathways that were opened, completed successfully, or unsuccessfully and the reason why, and any tools utilized to help support client needs. The complete process is shown in Fig 2.

The model to address social needs noted above was derived from the validated Agency for Healthcare Research and Quality (AHRQ) Pathways Community Hub model [32]. This comprehensive, evidence-based approach leverages the known impact of care coordination facilitated by a Community Health Worker (CHW) to complete social needs-related screenings and referrals to address identified needs via coordination of health and social services across multiple community settings [33–36]. Notably, the Pathways Community Hub model facilitates cross-sector integration by supporting data sharing and aligning payment models through reimbursement for completed referrals to community-based organizations (CBOs) [37,38]. Specifically, the HCGC Hub model consists of three features [39]: (1) A regional coordination entity that employs CHWs to assess the medical and social needs of vulnerable patients and connect them to community resources; (2) the CHWs initiate a “care pathway,” a defined action plan that describes how patient needs will be addressed, which is then recorded and tracked in an electronic database (S1 Table); (3) completion of each care pathway is linked to payment from healthcare payers (Medicaid-managed care plans and other community partners) based on specific performance benchmarks. A financial contract is attached to each standardized care pathway; when a care pathway is completed, a CHW must confirm that a measurable outcome (e.g., patient has received food) is obtained in order for the agency to receive payment (Fig 2).

### Data collection and measures

Biometric assessments were performed at baseline, 12 weeks, and 24 weeks. Data from participants included self-reported measures (sociodemographic and self-reported health history), survey data collected via REDCap, including the CMS Accountable Health Communities Health-Related Social Needs Screening Tool either onsite at the Recreation and Parks locations or at participant homes [30]. Biometric measurements, including blood pressure (mmHg), fasting cholesterol (mg/dl), fasting glucose (mg/dl), weight (lbs), and BMI were collected onsite at the Recreation and Parks locations and recorded in REDCap at each time point. The sociodemographic data included age, education, race, ethnicity, employment status, insurance status, and annual income. The self-reported health history included hypertension, diabetes, hyperlipidemia, and smoking status (I have never smoked, I currently smoke, I quit smoking > 1 year ago or I quit smoking ≤ 1 year ago), as well as medications for the aforementioned chronic conditions.

The survey data included the Diet History Questionnaire (DHQ) III [40]. The DHQ-III nutrient and food group database is based on a compilation of national 24-hour dietary recall data from the National Health and Nutrition Examination Surveys (NHANES). Prior research has shown the questionnaire is valid and reliable [41–43]. In the current evaluation, we calculated physical activity minutes per week using the validated moderate physical activity 2-question physical activity questionnaire within the CMS screening tool [44].

Biometric screenings were performed by trained healthcare staff, including nurses and physicians. Blood pressure was checked via an automated oscillometric sphygmomanometer (Omron 5 series) with two measurements performed after the participants were seated for 5 minutes and averaged. Weight was measured using a zeroed and calibrated Omron Body Composition Monitor and Scale (Model: HBF-514C). Height was measured via a tape measurer. BMI was calculated by multiplying weight (lbs) by 703 and then dividing by height squared (inch^2^). Blood total cholesterol and glucose were measured in the fasting state using the Cardio Check Silver® (Polymer Technology, Inc., Heath, OH, USA) device. All participants received individual results at baseline, 12, and 24 weeks.

#### Social needs outcome

The main outcome in this analysis was change in social needs at 12 and 24-weeks compared to baseline. Social needs were coded as a “1” if any social need was identified on the CMS Accountable Health Communities Health-Related Social Needs Screening Tool [30,31] and “0” if none were identified. The social needs in the analysis included: 1) unstable or unsafe living situation; 2) food insecurity; 3) lack of transportation; 4) challenges with utilities; 5) physical safety; 6) financial strain; and 7) employment. The safety score was calculated from the 4 question HITS short domestic violence screening tool (a component of the CMS screening tool) with Likert scale answers from “Never” to “Frequently” with scores ranging from 1 (Never) to 5 (Frequently) [45]. A score of 11 or more when the numerical values for answers to questions 7–10 were added showed that the person might not be safe and was coded as a “1,” as has been validated in women and men [45,46].

#### Cardiovascular health outcomes

The secondary outcome measure was change in LS7 cardiovascular health score (range 0–14) by baseline social needs. The LS7 cardiovascular health score was summed based on the individual LS7 metrics (glucose, cholesterol, blood pressure, BMI, physical activity, diet and smoking) categories of poor (0 points), intermediate (1) and ideal (2) cardiovascular health at baseline, 12 and 24 weeks (S2 Table), based on the AHA guidelines [47], as has been done previously [17,18,25]. Additionally, we developed a score using 6 components of the LS7 cardiovascular health score excluding diet (range 0–12) and 5 components excluding diet and physical activity (range 0–10). The 5 and 6 component scores were used in sensitivity analyses to confirm the robustness of the findings given that diet and physical activity were self-reported.

### Statistical analysis

Descriptive statistics were performed for all variables, including mean (standard deviation [SD]) for continuous variables and frequencies and percentages for categorical variables. These characteristics have been compared between participants with and without social needs using two-sample t-test for continuous variables and chi-squared or fisher’s exact test for categorical variables. Mixed effect logistic regressions (generalized mixed models) with random intercepts for each participant were used to examine the change of social need from baseline to 24 weeks.

**Fig 3:**
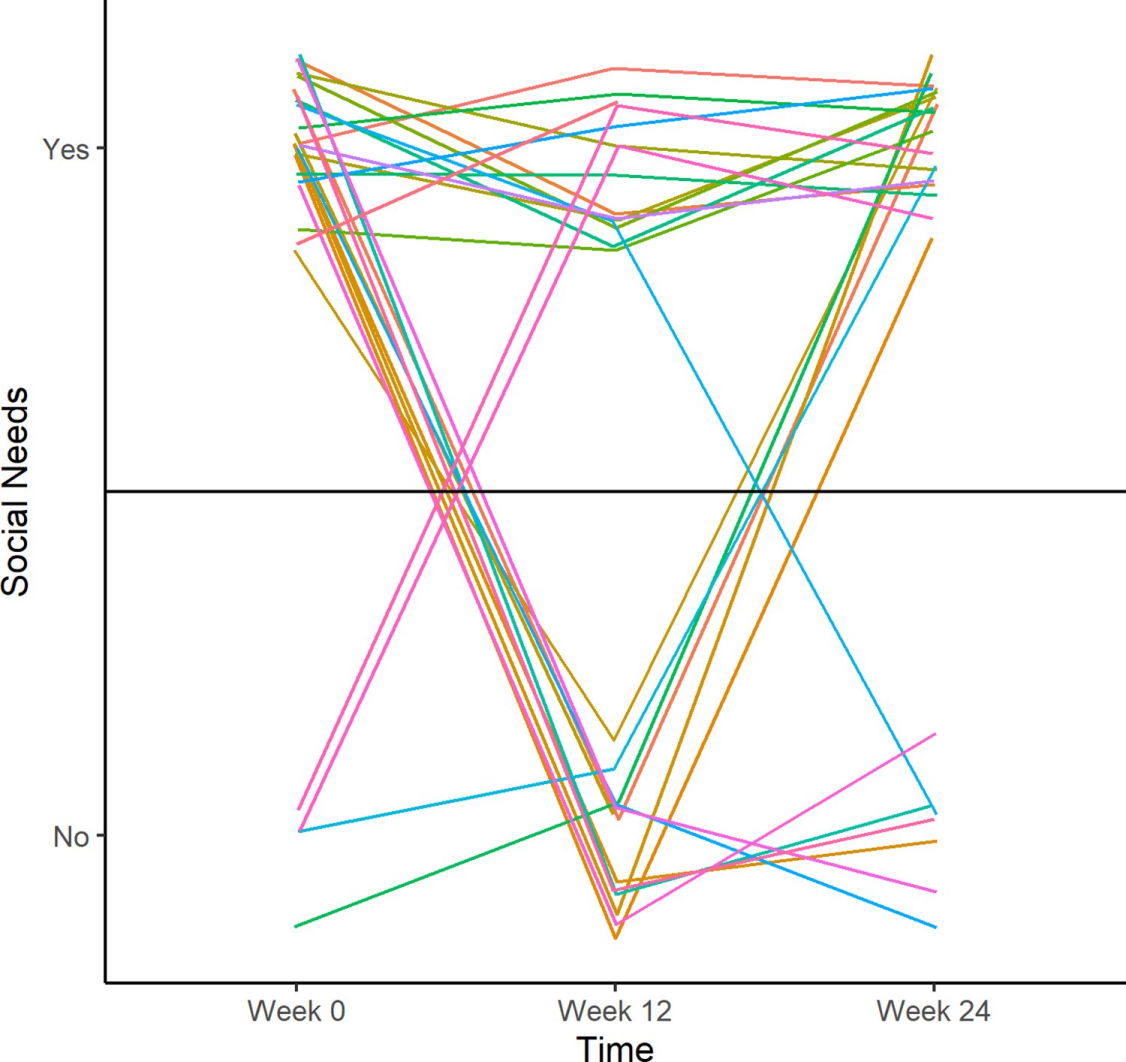
Non-medical health related social needs in black impact at 0, 12 and 24 Weeks. Lines represent the 44 participants that had social need(s) at some point during the study. Four had no social needs at baseline, then gained at least one social need as the study progressed. Forty had a social need at baseline. Thirteen of the 40 had no social needs by week 12, and one participant took until week 24 to resolve his social need(s). Six of the 13 that had social needs addressed by week 12 had regained social need(s) by week 24. The lines in the plot have random jitter added to allow individual participants to be distinguished.

Odds ratios (ORs) and corresponding 95% confidence intervals (CIs) were reported. The models were sequentially adjusted for: 1) age and; 2) age and education. Additionally, change in LS7 cardiovascular health was calculated using linear mixed models with random intercepts for each participant stratified by baseline social needs status. Sensitivity analyses were performed where each instance of a social need from the first 12 questions of the CMS survey were counted at each time point and listed in S3 Table. If a participant had one of those listed social needs at a time point, they were considered to have a social need at that time point. A separate data set was created that included only those men who had a social need at any time point (n = 44). A spaghetti plot showing change in social needs among these participants per time point is presented in Fig 3. Statistical significance for all analyses was defined as two-sided alpha < 0.05. Statistical analyses were performed using SAS 9.4 (SAS Institute, Inc.; Cary, North Carolina, USA) and R version 3.4.3 (R Foundation for Statistical Computing, Vienna, Austria).

## Results

Seventy-four Black men participated in the intervention, and 70 are included in the current analysis (Fig 1). Baseline demographic characteristics of Black Impact participants are shown in Table 1. The mean age of participants was 52.0 years (SD 10.5). All participants had a high school degree or equivalent and 43% had a college degree or higher level of education. The majority of participants were employed with private insurance (84.3% and 72.9%, respectively). The income of participants was heterogeneous, ranging from <$20,000 (5.7%) to ≥$75,000 (22.9%). LS7 cardiovascular health scores had a high proportion of participants in the poor range for blood pressure (47.1%), glucose (28.6%), body mass index (54.3%), and diet (40.0%). At baseline there was no difference in cardiovascular health scores stratified by social needs status (identified social need vs. not) using 5 (excluding diet and physical activity), 6 (excluding diet), or all 7 components of the cardiovascular health scores (Life’s Simple 7).

**Table 1:** Characteristics of participants in the black impact pilot study by baseline non-medical health related social needs at baseline.

Baseline Characteristics^a^^,^^b^	Overall (N = 70)	No Social Needs (N = 30)	Social Needs (N = 40)	p-value
**Age**	52.0 (10.5)	54.9 (9.29)	49.8 (11.0)	0.046
**Marital Status**				0.091^d^
Never Married	18 (25.7%)	4 (13.3%)	14 (35.0%)	
Married	37 (52.9%)	19 (63.3%)	18 (45.0%)	
Separated	1 (1.4%)	1 (3.3%)	0 (0%)	
Divorced	13 (18.6%)	5 (16.7%)	8 (20.0%)	
Widowed	1 (1.4%)	1 (3.3%)	0 (0%)	
**Number of Children**	3.03 (1.57)	3.30 (1.29)	2.83 (1.74)	0.213
**Annual Income**				<0.001^d^
<$20,000	4 (5.7%)	0 (0%)	4 (10.0%)	
$20,000-$49,999	19 (27.1%)	7 (23.3%)	12 (30.0%)	
$50,000-$74,999	21 (30.0%)	5 (16.7%)	16 (40.0%)	
≥$75,000	16 (22.9%)	15 (50.0%)	1 (2.5%)	
Missing	10 (14.3%)	3 (10.0%)	7 (17.5%)	
**Education**				0.640^d^
High School or equivalent	6 (8.6%)	2 (6.7%)	4 (10.0%)	
Vocational/Technical School (2 year)	7 (10.0%)	3 (10.0%)	4 (10.0%)	
Some College	27 (38.6%)	9 (30.0%)	18 (45.0%)	
College Graduate (4 year)	18 (25.7%)	9 (30.0%)	9 (22.5%)	
Master’s Degree (MS)	11 (15.7%)	6 (20.0%)	5 (12.5%)	
Professional Degree (MD,JD, etc.)	1 (1.4%)	1 (3.3%)	0 (0%)	
**Employed**				0.142
No	11 (15.7%)	2 (6.7%)	9 (22.5%)	
Yes	59 (84.3%)	28 (93.3%)	31 (77.5%)	
**Health Insurance**				0.418^d^
Private insurance	51 (72.9%)	25 (83.3%)	26 (65.0%)	
Medicaid/Medicare	6 (8.6%)	2 (6.7%)	4 (10.0%)	
Military insurance	4 (5.7%)	1 (3.3%)	3 (7.5%)	
No insurance	9 (12.9%)	2 (6.7%)	7 (17.5%)	
**Systolic Blood Pressure (mmHg)**	139 (20.2)	140 (16.2)	139 (23.0)	0.845
Missing	1 (1.4%)	0 (0%)	1 (2.5%)	
**Diastolic Blood Pressure (mmHg)**	87.5 (13.4)	88.8 (13.0)	86.5 (13.9)	0.477
Missing	1 (1.4%)	0 (0%)	1 (2.5%)	
**Blood Glucose (mg/dL)**	123 (53.3)	124 (43.6)	122 (60.1)	0.865
**Total Cholesterol (mg/dL)**	159 (44.5)	163 (45.0)	157 (44.6)	0.593
**Body Weight (lbs)**	237 (65.2)	240 (59.9)	235 (69.5)	0.740
**Body Mass Index (kg/m2)**	33.1 (7.57)	33.8 (7.45)	32.6 (7.72)	0.519
**Cholesterol Medications**				0.461
No	51 (72.9%)	20 (66.7%)	31 (77.5%)	
Yes	19 (27.1%)	10 (33.3%)	9 (22.5%)	
**Diabetes Medication**				0.494
No	53 (75.7%)	21 (70.0%)	32 (80.0%)	
Yes	17 (24.3%)	9 (30.0%)	8 (20.0%)	
**Anti-Hypertensive Medications**				0.809
No	35 (50.0%)	14 (46.7%)	21 (52.5%)	
Yes	35 (50.0%)	16 (53.3%)	19 (47.5%)	
**Life’s Simple 7 Score (0–14)** ^**c**^	7.48 (1.76)	7.54 (1.58)	7.44 (1.91)	0.834
Missing	10 (14.3%)	4 (13.3%)	6 (15.0%)	
**Life’s Simple 6 Score (0–12)** ^**c**^	6.78 (1.75)	6.73 (1.60)	6.82 (1.88)	0.839
Missing	1 (1.4%)	0 (0%)	1 (2.5%)	
**Life’s Simple 5 Score (0–10)** ^**c**^	5.33 (1.69)	5.20 (1.58)	5.44 (1.79)	0.570
Missing	1 (1.4%)	0 (0%)	1 (2.5%)	
**LS7 Body Mass Index**				0.202
Ideal	7 (10.0%)	1 (3.3%)	6 (15.0%)	
Intermediate	25 (35.7%)	13 (43.3%)	12 (30.0%)	
Poor	38 (54.3%)	16 (53.3%)	22 (55.0%)	
**LS7 Physical Activity**				0.567^d^
Ideal	36 (51.4%)	17 (56.7%)	19 (47.5%)	
Intermediate	29 (41.4%)	12 (40.0%)	17 (42.5%)	
Poor	5 (7.1%)	1 (3.3%)	4 (10.0%)	
**LS7 Blood Glucose**				0.633
Ideal	18 (25.7%)	6 (20.0%)	12 (30.0%)	
Intermediate	32 (45.7%)	15 (50.0%)	17 (42.5%)	
Poor	20 (28.6%)	9 (30.0%)	11 (27.5%)	
**LS7 Blood Pressure**				0.332^d^
Ideal	5 (7.1%)	1 (3.3%)	4 (10.0%)	
Intermediate	31 (44.3%)	12 (40.0%)	19 (47.5%)	
Poor	33 (47.1%)	17 (56.7%)	16 (40.0%)	
Missing	1 (1.4%)	0 (0%)	1 (2.5%)	
**LS7 Smoking Status**				0.125^d^
Ideal	58 (82.9%)	28 (93.3%)	30 (75.0%)	
Intermediate	2 (2.9%)	0 (0%)	2 (5.0%)	
Poor	10 (14.3%)	2 (6.7%)	8 (20.0%)	
**LS7 Cholesterol**				0.929^d^
Ideal	39 (55.7%)	16 (53.3%)	23 (57.5%)	
Intermediate	26 (37.1%)	12 (40.0%)	14 (35.0%)	
Poor	5 (7.1%)	2 (6.7%)	3 (7.5%)	
**LS7 Diet**				1.000^d^
Ideal	1 (1.4%)	0 (0%)	1 (2.5%)	
Intermediate	32 (45.7%)	14 (46.7%)	18 (45.0%)	
Poor	28 (40.0%)	12 (40.0%)	16 (40.0%)	
Missing	9 (12.9%)	4 (13.3%)	5 (12.5%)	

^a^ Mean (SD) or count (percentage) are listed, p-values calculated using chi-square (categorical variables), and two-sample t-test (parametric continuous variables).

^b^ n = 70 participants for age, blood glucose (mg/dL), total cholesterol (mg/dL), weight (lbs), BMI, medications, marital status, employment status, and number of children; 60 participants for income and LS7; and 69 participants for all other categories.

^c^ AHA = American Heart Association, LS7 = Life’s Simple 7, Cardiovascular Health recommendations were defined by AHA 2020 guidelines (see S1 Table).

^d^ Fisher exact test was used instead of chi-square for violations of the chi-square test due to low-frequency cells.

Forty out of the 70 men (57.1%) had an identified social need. Thirty-one of the 40 men were interested and referred to the HCGC Hub. Eight of the thirty-one men were enrolled into pathways including education, social services, medical referral, behavioral health, employment, and medical home pathways.

The longitudinal change in social needs is shown in Table 2. The 57.1% of participants with an identified social need at baseline decreased to 36.6% and 44.2% at 12 and 24 weeks, respectively. The odds of having a social need at week 12 and 24 were 67% lower (OR 0.33, 95% CI: 0.13, 0.85) and 50% lower (OR 0.50, 95% CI: 0.21, 1.16) than baseline. Given the study occurred during the COVID-19 pandemic we also evaluated social needs using 5 of the 7 social needs components excluding financial strain or employment. The results were similar with significant reductions in social needs at 12 weeks (p = 0.035) and a trend at 24 weeks (p = 0.13). Among the 7 individual social needs the majority showed trends in the direction of improvement except for employment with a numerically higher proportion (non-significant) of participants indicating a desire for “help finding work” or “help keeping work”.

**Table 2:** Longitudinal change in non-medical health-related social needs at baseline, Week 12 and Week 24.

Social Needs	Intervention Week	Participants	Participants with Social Needs (n)	Participants with Social Needs (%)	Model 0—Unadjusted			Model 1—Age			Model 2 –Age & Education		
					Odds Ratio	95% CI	p-value	Odds Ratio	95% CI	p-value	Odds Ratio	95% CI	p-value
Non-medical health-related social needs*	Baseline	70	40	57.14%									
	week12–baseline	41	15	36.59%	0.32	(0.13, 0.81)	0.017	0.33	(0.13, 0.82)	0.018	0.33	(0.13, 0.85)	0.021
	week24–baseline	52	23	44.23%	0.50	(0.22, 1.13)	0.096	0.50	(0.22, 1.14)	0.100	0.50	(0.21, 1.16)	0.104
Non-medical health-related social needs excluding Financial strain or Employment*	Baseline	70	27	38.57%									
	week12–baseline	41	9	21.95%	0.29	(0.10, 0.88)	0.030	0.29	(0.10, 0.90)	0.032	0.30	(0.10, 0.91)	0.035
	week24–baseline	52	15	28.85%	0.47	(0.18, 1.23)	0.125	0.47	(0.18, 1.25)	0.130	0.47	(0.18, 1.25)	0.131
Living situation	Baseline	70	19	27.14%									
	week12–baseline	41	7	17.07%	0.44	(0.15, 1.32)	0.143	0.43	(0.14, 1.30)	0.132	0.42	(0.14, 1.30)	0.133
	week24–baseline	52	9	17.31%	0.47	(0.17, 1.26)	0.131	0.46	(0.17, 1.24)	0.123	0.45	(0.17, 1.24)	0.122
Food Security	Baseline	70	10	14.29%									
	week12–baseline	41	5	12.20%	0.86	(0.23, 3.21)	0.817	0.90	(0.24, 3.41)	0.874	0.94	(0.24, 3.74)	0.935
	week24–baseline	52	7	13.46%	0.80	(0.25, 2.63)	0.716	0.82	(0.24, 2.72)	0.740	0.81	(0.24, 2.79)	0.741
Transportation	Baseline	70	9	12.86%									
	week12–baseline	41	3	7.32%	0.35	(0.07, 1.74)	0.198	0.34	(0.07, 1.70)	0.187	0.33	(0.06, 1.72)	0.188
	week24–baseline	52	3	5.77%	0.31	(0.06, 1.46)	0.137	0.29	(0.06, 1.43)	0.129	0.29	(0.06, 1.43)	0.127
Utilities	Baseline	70	10	14.29%									
	week12–baseline	41	6	14.63%	0.94	(0.24, 3.61)	0.923	0.97	(0.25, 3.81)	0.969	1.04	(0.25, 4.31)	0.952
	week24–baseline	52	8	15.38%	0.92	(0.27, 3.17)	0.897	0.94	(0.27, 3.31)	0.924	0.95	(0.26, 3.47)	0.936
Safety	Baseline	70	1	1.43%									
	week12–baseline	41	0	0%	n/a			n/a			n/a		
	week24–baseline	52	1	1.92%	n/a			n/a			n/a		
Financial strain	Baseline	70	22	31.43%									
	week12–baseline	41	7	17.07%	0.37	(0.12, 1.08)	0.069	0.36	(0.12, 1.07)	0.065	0.36	(0.12, 1.10)	0.073
	week24–baseline	52	15	28.85%	0.77	(0.32, 1.87)	0.562	0.76	(0.31, 1.85)	0.543	0.76	(0.31, 1.88)	0.551
Employment	Baseline	70	12	17.14%									
	week12–baseline	41	10	24.39%	1.54	(0.48, 4.98)	0.468	1.56	(0.48, 5.08)	0.460	1.72	(0.50, 5.92)	0.388
	week24–baseline	52	14	26.92%	1.92	(0.66, 5.58)	0.228	1.95	(0.66, 5.70)	0.223	2.10	(0.69, 6.36)	0.187

Mixed effect logistic regressions (generalized mixed models) with random intercepts were used to explore the change of outcome measures across time. Odds ratios between each time point with baseline and 95% confidence intervals (CI) and p-values are reported.

Example Interpretation: In Week 12, participants had a 67% lower odds of reporting a non-medical health-related social need (OR 0.33, 95%CI: 0.13–0.85, p = 0.021 compared with baseline in age and education adjusted models.

*Social needs were coded as a “1” if any social need was identified on the survey and “0” if none were identified. The social needs in the analysis included: 1) unstable or unsafe living situation; 2) food insecurity; 3) lack of transportation; 4) challenges with utilities; 5) physical safety; 6) financial strain; and 7) employment. The safety score was calculated from 4 questions with Likert scale answers from “Never” to “Frequently” with scores ranging from 1 (Never) to 5 (Frequently). A score of 11 or more when the numerical values for answers to questions 7–10 were added showed that the person might not be safe and was coded as a “1”.

Table 3 shows the longitudinal change of cardiovascular health stratified by baseline social needs. Cardiovascular health scores improved by 0.94 points (p = 0.013) and 0.87 points (p = 0.022) in the group without and with social needs, respectively. No differential effect by baseline social needs status existed (interaction p-value for the interaction effect between social needs and time was p = 0.895).

**Table 3:** Longitudinal change of cardiovascular health scores at Week 12 and Week 24 stratified by baseline non-medical health-related social needs status.

Social Needs* (Yes/No)	Intervention Week	Number of Participants	Estimate Model 0	95% CI	p-value	Interaction Term	Estimate Model 1	95% CI	p-value	Interaction Term	Estimate Model 2	95% CI	p-value	Interaction Term
Life’s Simple 7 (LS7) Cardiovascular Health (CVH) Scores														
No	Week 1—Referent	26	7.44	(6.75, 8.13)		0.898	7.44	(6.74, 8.13)		0.891	7.42	(6.73, 8.11)		0.895
	Week 12 vs. Week 01	19	0.69	(-0.06, 1.45)	0.070		0.69	(-0.06, 1.44)	0.072		0.70	(-0.05, 1.45)	0.069	
	Week 24 vs. Week 01	22	0.93	(0.20, 1.66)	0.013		0.93	(0.20, 1.66)	0.013		0.94	(0.21, 1.67)	0.013	
Yes	Week 1 –Referent	34	7.45	(6.82, 8.07)			7.44	(6.80, 8.07)			7.53	(6.89, 8.17)		
	Week 12 vs. Week 01	12	0.92	(0.06, 1.78)	0.037		0.93	(0.06, 1.79)	0.036		0.91	(0.04, 1.77)	0.040	
	Week 24 vs. Week 01	18	0.89	(0.15, 1.63)	0.019		0.90	(0.16, 1.64)	0.018		0.87	(0.13, 1.61)	0.022	

To explore the change of cardiovascular health scores stratified by baseline non-medical health-related social needs status, an interaction term was inserted (Social Needs Status * time) in the linear mixed models. Differences of cardiovascular health scores between each time point with baseline and 95% confidence intervals (CI) and p-values are reported by participants with or without social needs, respectively.

Example Interpretation: There were no significant interaction effect between social needs and time for all the models. For example, in Week 24 Model 0, LS7 Score (0–14) was 0.93 points higher (difference = 0.93, 95% CI 0.20, 1.66 [p = 0.013]) for individuals without social needs and 0.89 points higher (difference = 0.89, 95% CI 0.15, 1.63 [p = 0.019]) among participants with social needs compared with baseline in the unadjusted model.

Model 0: Unadjusted, Model 1: Adjusted for Age, Model 2: Adjusted for Age and Education.

In sensitivity analyses among participants with social needs, among the 40 participants with social needs at baseline 13 had the social need resolved by week 12, but 4 of the 13 noted a new social need by week 24 (Fig 3).

## Discussion

In this novel, 24-week CBPR lifestyle intervention that addressed social needs through screening and service coordination in Black men, a majority of men had social needs at baseline and were interested in addressing them through a referral program. Reductions in social needs were seen at 12 and 24 weeks. Social needs status at baseline was not associated with baseline cardiovascular health scores nor change in cardiovascular health scores at 12 and 24 weeks. To our knowledge this is the first study focused on addressing a complete suite of cardiovascular risk factors through a program that includes a lifestyle intervention, along with assessing and addressing social needs in Black men.

### Addressing social needs in a comprehensive intervention

The referrals through the HCGC Hub model facilitated cross-sector integration between the Black Impact program and CHWs to address social needs [37,38]. The model also supported data sharing and aligned payment models through reimbursement for completed referrals to CBOs. Previous programs have addressed social needs through social workers, navigators, CHWs, advocates or referral-based programs in healthcare systems and other settings [7,48,49]. Programming solely focusing on addressing social needs without other components has been systematically reviewed [7]. The majority of interventions reported successfully identifying unmet social needs and referring to clinic and community resources [7]. There was wide heterogeneity in the uptake of the referrals, but generally studies show improvement in social needs [7]. Additionally, previous analyses of Pathways Community Hub models have shown that counties with broad networks of community-based services focused on care coordination to address the social needs of older adults had lower readmission rates and less avoidable nursing home care [50]. Likewise, a population-level analysis of cross-sector collaborations demonstrated lower mortality associated with cardiovascular disease, diabetes, and influenza [51]. The Black Impact study is the first study to our knowledge to address social needs as part of a comprehensive, community-based intervention founded in improving cardiovascular health in Black men.

### Social needs and life’s simple 7

Limited data exists on the impact of social needs on LS7. The study team hypothesized that participants with social needs would have worse cardiovascular health per the AHA LS7 measures at baseline. The hypothesis was not supported by our findings. The majority of the extant literature examines the relationship between socioeconomic status and LS7. In Black adults in the Jackson Heart Study, higher individual income, neighborhood socioeconomic status and education were associated with higher LS7 cardiovascular health scores, although sex-stratified findings were not reported and two-thirds of Jackson Heart Study participants are women [13]. Thus, it is unclear if these findings are consistent in Black men. In Black men participating in US AAMWA Walks there was no association of higher levels of education or employment status with six components (excluding diet) of the LS7 cardiovascular health scores, but were positive associations with income and insurance [14]. Caleyahetta et al. showed that a higher cumulative risk score summing four socioeconomic status measures (low family income, low education level, minority race, and single-living status) was associated with lower attainment of LS7 [52].

In terms of specific social needs, there are studies evaluating the relationship of social needs, most commonly housing, food insecurity, and financial stress with cardiovascular risk factors or chronic disease [53–55]. There are limited studies evaluating social needs and LS7. In a weight loss study in Louisiana (two-thirds Black participants), the mean LS7 total score was not significantly different by food security status at baseline [56]. Additionally, there was no association of food insecurity with adiposity in men [56]. Discordant with our study, food insecurity was associated with lower prevalence of “good” (LS7 ideal and intermediate vs. poor) cardiovascular health among majority White (85%) individuals in Wisconsin [57]. Contrary to the authors hypotheses, food insecure individuals were more likely to have ideal blood pressure and total cholesterol [57]. Among middle age and older female health professional majority (95%) White women, the number of financial stressors was associated with lower ideal cardiovascular health [58]. Lack of housing has been associated with higher cardiovascular disease risk, but there are no studies assessing housing security and/or quality and LS7, as most large cardiovascular cohorts and national data sources do not include individuals with significant housing insecurity or homelessness [59]. Thus, the findings of Black Impact showing no association of social needs and Life’s Simple 7 at baseline are an important contribution to the limited extant literature. The lack of association of social needs with LS7 in Black Impact may be due to multiple factors as previously noted by Azap et al. including “John Henryism”, allostatic load from multiple stresses including racism and discrimination, discrimination and bias in the healthcare setting leading to medical mistrust, and inequities in wealth, such that those without social needs, may still have difficulty attaining high levels of cardiovascular health [14,29]. Further studies addressing the role of social needs in LS7 cardiovascular health are a critical area of inquiry to discern the role and mechanisms of social needs in cardiovascular risk factors particularly among racial/ethnic minority sex groups.

### Social needs interventions and life’s simple 7

The authors hypothesized that even with addressing social needs as part of the Black Impact intervention, participants with baseline social needs would have less improvements in cardiovascular health scores. Importantly, in the study there was no difference in improvement in cardiovascular health scores across 12 and 24 weeks, suggesting that improvements in social needs and addressing cardiovascular health through physical activity and health education in a community-setting may be a potential strategy to advance cardiovascular health irrespective of social needs.

There are limited studies to which to compare the Black Impact study, due to the novel nature of the community-based participatory research intervention in Black men. Interventions addressing social needs and cardiovascular risk factors have mostly focused on the healthcare setting. Overall, studies reporting health, utilization, or cost outcomes report mixed results [7]. A prominent study by Berkowitz, et al. used advocates in the healthcare setting to help individuals obtain resources across multiple social needs and showed reductions in blood pressure and cholesterol but not glycemia in individuals engaged in healthcare with cardiometabolic diseases [48]. Kangovi et al. studied goal setting vs. goal setting and a CHW to improve glycemia, blood pressure, obesity, or smoking in 302 individuals who were predominantly Black (95%) and female (75%) over 6-months [60]. While none of the individual categories were significantly different for goal setting vs. goal setting/CHW, there was a trend (p = 0.08) towards overall greater improvements in the outcomes (A1c, systolic blood pressure, BMI, or smoking) with the addition of the CHW [60]. The study team performed a follow-up study in 592 individuals with similar characteristics over 9 months and found no difference in changes in self-rated physical health, mental health nor a combination of A1c, systolic blood pressure, BMI, or smoking (p = 0.21) [61].

In a systematic review by Gottlieb et al, the authors found 81 studies where social needs were addressed as part of comprehensive interventions [7]. Only three of these studies had components of LS7 as outcomes and none had all 7 factors. Watt et al, performed a primary care based early childhood intervention in low-income Hispanic pregnant women. The program provided vouchers for fruits and vegetables from the local farmers’ market, nutrition classes, cooking classes, and lactation counseling. Women in the intervention compared to control had significant improvements in diet, exercise, and depression (p≤0.05) [62]. Loskutova et al, examined telephone-based nonprofessional patient navigation to promote linkages between the primary care provider and community programs in 179 patients with or at risk for diabetes. Two patient navigators provided services over the phone, including assessment of needs, barriers and limitations, motivational interviewing and a suggestion of 2 to 3 community programs with an average of 6 calls per patient. In pre-post analyses they showed a reduction in hemoglobin A1c (7.8% vs 7.2%, P = 0.001) in those with diabetes and improvement in patient self-efficacy. They found no change in fasting glucose, BMI, total cholesterol, low-density lipoprotein, high-density lipoprotein, or triglycerides [63]. At Intermountain Healthcare, a generalist model of chronic disease management was formulated, with care managers located within multi-payer primary care clinics collaborating with physicians, patients, and other members of a primary care team to improve patient outcomes. In patients with diabetes, they found a greater reduction in A1c compared to controls over 1 year (8.0% to 7.4% [intervention] compared to 7.7% to 7.5% [control, p<0.001]) [64].

In comparison, the nearly 1-point increase in cardiovascular health score from baseline to 24-weeks in Black Impact was a large improvement in cardiovascular health, considering a 1-point higher cardiovascular health score is associated with an 18% and 19% lower odds of stroke and myocardial infarction, respectively and an 11% and 19% lower risk of all-cause and cardiovascular mortality [25]. Additionally, there were improvements in individual components including body mass index, systolic blood pressure, fasting glucose, total cholesterol and dietary intake [25].

### Strengths/Limitations

The strengths of our study include: 1) a focus on an understudied population with large disparities in cardiovascular health and mortality; 2) community engagement framework for CBPR that recognized the importance of addressing social needs in Black Impact; 3) collaborations across a number of organizations to screen and address social needs through the evidenced-based Pathways Community Hub model; and 4) the use of trained health professionals using evidence-based approaches for biometric data collection. Despite these strengths, the study should be considered in light of some limitations. First, the study was not randomized due to: 1) no previous test of intervention feasibility and acceptability; and 2) concerns raised from community members in regards to not receiving a potentially beneficial intervention (albeit, novel and not previously tested). A second limitation is the lack of a control group. Our findings may be influenced by regression toward the mean, but this is unlikely given the difficulty in addressing social needs without a supportive system. The effect estimates generated from the study are being used to plan a powered, randomized, wait-list controlled intervention. Third, even with our sociodemographically diverse cohort, the Black Impact participants may not be representative of other populations of Black men. Fourth, only 8 of the 31 men referred to the HGCG Hub were enrolled in a pathway. For the 23 non-enrolled men they were given paper resources but were not interested in being enrolled in a pathway. In future iterations of Black Impact, the study team will consider performing further education with participants regarding the importance of addressing social needs and the benefit of enrolling in pathways. Additionally, we have discussed having the CHW come to the Black Impact study site to meet with participants and build relationships. Increased enrollment in pathways may lead to even greater reductions in social needs over the course of the intervention. Lastly, our study was performed during the COVID-19 pandemic and participants may have different social needs in a non-pandemic setting. Notably, the lack of worse cardiovascular health at baseline among participants with vs. without social needs may have been due to impacts of the COVID-19 pandemic increasing social needs and worsening cardiovascular health due to pandemic restrictions (e.g. closure of gyms, food shortages, etc.) and difficulty accessing preventive medical care.

## Conclusion

To our knowledge this is the first study to show improvements in social needs as a component of a comprehensive lifestyle intervention in Black men. As part of the community engagement and co-design of the intervention using the PETAL framework for CBPR, addressing social needs was determined to be a key component. There was no evidence of baseline differences in LS7 by social needs, and the group with baseline social needs had similar improvements in cardiovascular health. In future iterations of Black Impact, it will be important to include an arm of the study that does not address social needs to determine the necessity of addressing social needs for LS7 improvement. Further research to evaluate how the dose and timing of addressing social needs may impact physical, mental health and quality of life outcomes is also warranted. Addressing social needs in Black men is an attainable goal through a multi-component intervention and may help individuals with social needs improve cardiovascular health.

## Supporting information

S1 Checklist (DOC)

S1 Table Core pathways available through the central ohio pathways hub model. (DOCX)

S2 Table American heart association definitions of poor, intermediate, and ideal cardiovascular health^a^. ^a^ Adapted from The American Heart Association’s Strategic Planning Task Force and Statistical Committee 2020 Guidelines [47]. ^b^ Adapted from The American Heart Association’s Strategic Planning Task Force and Statistical Committee 2020 Guidelines: Fruits and vegetables ≥4·5 cups/day, fish ≥two 3.5 ounce servings per week (non-fried), fiber-rich whole grains ≥ three 1 ounce-equivalent servings/day, sodium <1500 mg/day, and sugar-sweetened beverages ≤ 1884 kJ (36 ounces)/week [47].
(DOCX)

S3 Table Counts for individual social needs over time. Each instance of each social need from the first 12 questions of the CMS survey were counted at each time point and listed here.
(DOCX)

S1 File (PDF)
