# Supplementary material for: Addressing non-medical health-related social needs through a community-based lifestyle intervention during the COVID-19 pandemic: The Black Impact program
Source: PLoS One. 2023 Mar 9;18(3):e0282103. doi: 10.1371/journal.pone.0282103 (PMC9997965; doi:10.1371/journal.pone.0282103)
Supplement: S1 Table — (DOCX) [file pone.0282103.s002.docx]

Supplemental Table 1. Core Pathways Available Through the Central Ohio Pathways Hub Model

| **20 Core Pathways available through the Central Ohio Pathways Hub Model** | |
| --- | --- |
| Adult Education | Behavioral Health Referral |
| Employment | Developmental Screening |
| Health Insurance | Developmental Referral |
| Housing | Education |
| Medical Home | Family Planning |
| Medical Referral | Immunization Screening |
| Medication Assessment | Immunization Referral |
| Medication Management | Lead Screening |
| Smoking Cessation | Pregnancy |
| **Social Service Referral*** | Postpartum |

| ***Social Service Referral Pathway Referrals in the Columbus Pathways Hub Model** | |
| --- | --- |
| Child care services | Housing services –housing resource (not on Housing PW) |
| Child development services (Part C, Help Me Grow, Head Start) | Identification services (birth certificate, driver’s license, ID, etc.) |
| Child or elder abuse services | Intimate partner violence support services |
| Clothing – ongoing resource for clothing | Legal services |
| Citizenship – resource to obtain citizenship | Literacy – intervention and educational services |
| Day care/respite services | Medical debt support |
| Educational services and supports (not using Adult Learning PW) | Parenting education classes and support |
| Employment –employment resource (not on Employment PW) | Phone – resource to obtain phone services |
| Family crisis services (emergency shelter, red cross, etc.) | Safety equipment – (Examples: cribs, safety equipment for elders, car seats, locked cabinets for guns, bike helmets, fire extinguisher) |
| Fatherhood program and support services | Translation services – ongoing resource for translation services |
| Financial support – resource to financially assist with identified risk factor | Transportation – ongoing resource for transportation |
| Food stability – ongoing resource for food stability | Utilities – ongoing resource for utility support |
| Household items, including furniture |  |
