# Supplementary material for: Addressing non-medical health-related social needs through a community-based lifestyle intervention during the COVID-19 pandemic: The Black Impact program
Source: PLoS One. 2023 Mar 9;18(3):e0282103. doi: 10.1371/journal.pone.0282103 (PMC9997965; doi:10.1371/journal.pone.0282103)
Supplement: S2 Table — a Adapted from The American Heart Association’s Strategic Planning Task Force and Statistical Committee 2020 Guidelines [47]. b Adapted from The American Heart Association’s Strategic Planning Task Force and Statistical Committee 2020 Guidelines: Fruits and vegetables ≥4·5 cups/day, fish ≥two 3.5 ounce servings per week (non-fried), fiber-rich whole grains ≥ three 1 ounce-equivalent servings/day, sodium <1500 mg/day, and sugar-sweetened beverages ≤ 1884 kJ (36 ounces)/week [47]. (DOCX) [file pone.0282103.s003.docx]

Supplemental Table 2. American Heart Association Definitions of Poor, Intermediate, and Ideal Cardiovascular Health^a^

| Goal/Metric | Poor health | Intermediate health | Ideal health |
| --- | --- | --- | --- |
| Current smoking | Yes | Former ≤ 12 months | Never or quit >12 months |
| Total cholesterol | ≥ 240 mg/dl | 200-239 mg/dl  or treated to goal | <200 mg/dl |
| Blood pressure | SBP ≥140  or  DBP ≥90 mmHg | SBP 120-139  or DBP 80-89 mmHg  or treated to goal | <120/<80 mmHg |
| Body mass index | ≥30 kg/m^2^ | 25-29.9 kg/m^2^ | <25 kg/m^2^ |
| Physical activity | None | 1–149 min/wk moderate intensity  or  1–74 min/wk vigorous intensity | ≥150 min/wk moderate intensity  or  ≥ 75 min/wk vigorous intensity |
| Healthy diet score^b^ | 0-1 components | 2-3 components | 4-5 components |
| Fasting glucose | ≥126 mg/dl | 100-125 mg/dl  or treated to goal | <100 mg/dl |

Supplemental Table 2 Legend:

^a^ Adapted from The American Heart Association’s Strategic Planning Task Force and Statistical Committee 2020 Guidelines [47]

^b^ Adapted from The American Heart Association’s Strategic Planning Task Force and Statistical Committee 2020 Guidelines:Fruits and vegetables ≥4·5 cups/day, fish ≥two 3.5 ounce servings per week (non-fried), fiber-rich whole grains ≥ three 1 ounce-equivalent servings/day, sodium <1500 mg/day, and sugar-sweetened beverages ≤ 1884 kJ (36 ounces)/week [47].
