# Supplementary material for: Addressing non-medical health-related social needs through a community-based lifestyle intervention during the COVID-19 pandemic: The Black Impact program
Source: PLoS One. 2023 Mar 9;18(3):e0282103. doi: 10.1371/journal.pone.0282103 (PMC9997965; doi:10.1371/journal.pone.0282103)
Supplement: S3 Table — Each instance of each social need from the first 12 questions of the CMS survey were counted at each time point and listed here. (DOCX) [file pone.0282103.s004.docx]

Supplemental Table 3. Counts for Individual Social Needs over Time

| **Social Need** | **Week 0 (n=44)** | **Week 12 (n=28)** | **Week 24 (n=36)** |
| --- | --- | --- | --- |
| Living Worry | 1 | 1 | 1 |
| Pests | 8 | 4 | 2 |
| Mold | 4 | 3 | 3 |
| Lead | 1 | 0 | 1 |
| Heat | 2 | 2 | 1 |
| Oven/Stove | 1 | 0 | 1 |
| Smoke Detectors | 4 | 2 | 4 |
| Water Leaks | 5 | 4 | 5 |
| Food Worry | 0 | 0 | 1 |
| Food Ran Out | 7 | 4 | 7 |
| Transportation | 9 | 3 | 3 |
| Utilities | 10 | 6 | 8 |
| Safety | 1 | 0 | 1 |
| Financial | 22 | 7 | 15 |
| Help Keeping Work | 4 | 2 | 3 |
| Help Finding Work | 8 | 8 | 11 |
|  |  |  |  |
| Total Social Needs | 87 | 46 | 67 |

Supplemental Table 3 Legend: Each instance of each social need from the first 12 questions of the CMS survey were counted at each time point and listed here.
