# Supplementary material for: Addressing non-medical health-related social needs through a community-based lifestyle intervention during the COVID-19 pandemic: The Black Impact program
Source: PLoS One. 2023 Mar 9;18(3):e0282103. doi: 10.1371/journal.pone.0282103 (PMC9997965; doi:10.1371/journal.pone.0282103)
Supplement: S1 File — (PDF) [file pone.0282103.s005.pdf]

Research Protocol:
Creating Healthier Communities – AAMWI & OSU

Creating Healthier Communities through Meaningful Partnerships:
A Model from the National African American Male Wellness Initiative – OSU Partnership

Darrell M. Gray, II, MD, MPH, Timiya Nolan, PhD, APRN-CNP, James Odei, PhD, Joshua J. Joseph, MD

I. Objectives

Primary Objective: Test feasibility and acceptability of a 24-week community-based lifestyle intervention to improve cardiovascular health among African American males.

Secondary Objectives: 1) Navigate participants to establish primary care and address social and patient activation needs that present barriers to wellness; 2) Examine changes in cardiovascular health as per American Heart Association Life's Simple 7 Metrics including blood pressure, cholesterol, glucose, smoking, body mass index, physical activity and dietary intake; and 3) Increase African American male participation in clinical trials.

II. Background and Rationale

In 2010, the American Heart Association (AHA) published 2020 impact goals for cardiovascular health promotion with the aim of improving cardiovascular health and reducing deaths from cardiovascular disease (CVD) and stroke by 20% by the year 2020, which we have modified as shown in Figure 1 for use in the African American Male Wellness Initiative (AAMWI) walk.[1]

Goal/Metric		Poor health	Intermediate health	Ideal health
Current smoking		Yes	--	None
Total cholesterol		≥ 240 mg/dl	200-239 mg/dl or treated to goal	<200 mg/dl
Blood pressure		SBP ≥140 or DBP ≥90 mmHg	SBP 120-139 or DBP 80-89 mmHg or treated to goal	<120/<80 mmHg
Body mass index		≥30 kg/m^2	25-29.9 kg/m^2	<25 kg/m^2
Physical activity		Exercise < 3 times per week	--	Exercise ≥ 3 times per week
Healthy diet		--	--	--
Glucose	Fasting	≥126 mg/dl	100-125 mg/dl or treated to goal	<100 mg/dl
	Random	≥200 mg/dl	140-199 mg/dl	< 140 mg/dl

Figure 1. Life's Simple 7 Goals for Cardiovascular Health (adapted for AAMWI)

Higher attainment of ideal cardiovascular health, indicated by the Life's Simple 7 (LS7), has been associated with lower risk of cardiovascular disease, type 2 diabetes (diabetes), cancer and mortality among all races/ethnicities.[2–4] Nationally, African American (AA) men have the lowest levels of cardiovascular health. Analysis of data collected at the 2017 AAMWI walk shows that only 36% of screened participants had ≥ 4 ideal cardiovascular health measures defined as: non-smoker, exercising 3 or more days per week, blood pressure ≤ 120 / 80 mmHg, total cholesterol < 200 mg/dl, fasting glucose < 100 mmHg, or body mass index < 25 kg/m^2. Notably, 54% of individuals had hypertension, 44% of individuals had a body mass index in the obese range, 13% of

individuals had diabetes. The rates of LS7 seen in this population present a tremendous opportunity and critical need for improvement. Our prior research indicates that 64% (~544 in 2017) of AA men screened at the annual AAMWI walk have either poor (0-1 ideal CVH metrics) or average (2-3 ideal CVH metrics) cardiovascular health, which places them at higher risk for diabetes, CVD and cancer. For instance, 6 out of 10 new cases of diabetes are due to not having ≥ 4 ideal cardiovascular health metrics.[2] Community and provider level access and engagement to activate black men to embrace wellness and improve LS7 may lead to long-term reductions in diabetes, CVD and cancer, thus reducing disparities and improving health equity. Additionally, the lack of inclusion of AA men in clinical trials is a problem in Central Ohio and nationally that prevents the generalizability of many discoveries for the prevention and treatment of chronic diseases to AA men. Thus, there is a critical need for targeted programs to increase engagement of AA men in clinical trials.

The current efforts of the AAMWI have increased awareness of LS7 but have not led to sustained improvements in LS7 among AA men over time. For instance, from 2015-2017, ideal cardiovascular health metrics including: not smoking (ideal) decreased from 89% to 87%, ideal blood pressure decreased from 35% to 27%, ideal body mass index decreased from 22% to 19%, ideal cholesterol decreased from 81% to 69%. These sobering statistics partly reflect local and nationwide trends in AA cardiovascular health and the growing breadth of the AAMWI, which is attracting individuals from disparate areas and neighborhoods, which often have lower levels of health due to the impact of the social determinants of health. A recent analysis in Ohio, that length and quality of life for AAs in Ohio is far worse than other racial/ethnic groups and is even worse than the total population in the lowest ranked county in Ohio.[5] These findings demonstrate the critical importance for novel targeted programming to improve LS7 and are the basis for this study.

Rationale

The overarching goal is to develop a sustainable, community-based model for activating AA men, who are at-risk for poor outcomes from cardiometabolic diseases and cancer, to embrace wellness, increase attainment of cardiovascular health through LS7, and engage in clinical trials. We anticipate this being a flexible model that can be adapted to other populations across Ohio and beyond. We have presented the data that forms the basis of our rationale in peer-reviewed conferences and are concluding a systematic review of community-based interventions that informed the development of our novel intervention.

Research Structure:

In this single-arm pilot program,[6] we will enroll 100 AA male participants from the annual AAMWI walk/health fair who have poor or average cardiovascular health (< 4 LS7 metrics in the ideal range) with the goal of accomplishing aims exhibited in Figure 2. Our intervention involves two components: a community health worker (CHW) and peer team. Each participant will be assigned to a CHW and grouped into a team of 10 participants based on participant proximity to a central meeting location (total 10 teams). The CHW will assist in activating individual participants to engage in primary care, social support services, and enrollment in ResearchMatch. Within the teams, health coaches will assist the study team in implementing the community-based lifestyle intervention spanning LS7 principles and ResearchMatch, as shown in Figure 2 below.

Research Protocol:
Creating Healthier Communities – AAMWI & OSU

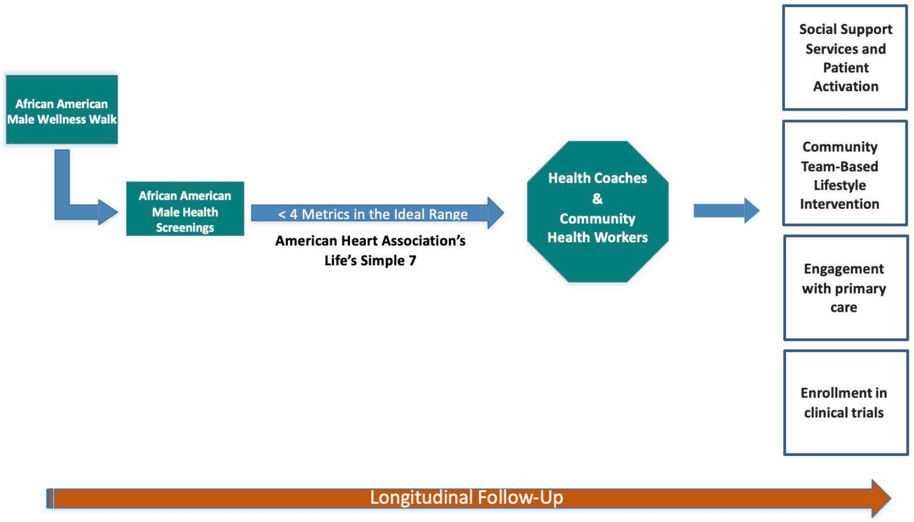

Figure 2. African American Male Wellness Initiative -OSU Partnership Intervention Model.

Instructors:
Each team will also be assigned four health coaches, two medical students, one undergraduate student and one nursing student, all of whom are identified from a database of volunteers from the OSU chapters of the Student National Medical Association (SNMA), Minority Association of Pre-Medical Students (MAPS) and Nursing Students Promoting Initiatives Reinforcing Equity (NSPIRE). Medical students will lead the student volunteer teams given their OSU College of Medicine training in health coaching. All instructors will have basic competency of curriculum and protocol.

Setting:
Teams will be assigned based on proximity of participants to a central meeting location, which will be community-based including Columbus Recreation and Parks centers or YMCAs.

Sample
Inclusion Criteria: 1) African American men (self-report); 2) Adult age 18 years or older. 3) Poor or average cardiovascular health (< 4 LS7 metrics in the ideal range); 4) Participant is appropriate for group setting.

Exclusion Criteria: 1) Non-English speaking; 2) Living outside of the Metro Columbus Area; 3) Unable to perform physical activity

Detailed Procedures:

Recruitment:
We will enroll 100 AA men that attend the annual AAMWI walk who have completed screenings offered by the AAMWI and who have poor or average cardiovascular health (< 4 LS7 metrics in the ideal range). Our team has had a relationship with the AAMWI for greater than three years. We will start recruitment at the AAMWI walk in August 2019. The AAMWI's screening takes place at the walk location. The AAMWI's established screening identifies individuals with suboptimal cardiovascular health who will be

directed to study team members including Dr. Darrell Gray, Dr. Timiya Nolan and Dr. Joshua Joseph at the event. The study team will discuss potential participation in the study. Potential participants who are unable to be consented may be contacted by telephone and email to setup a time for discussion of the study and potential consent.

Of note, approximately two-thirds of AAMWI participants from 2016-2018 meet eligibility criteria. With an annual screening rate of 500-700 participants, this yields a total of 1,320 to 1,848 participants that are eligible for participation in this study.

Randomization:
This is a single-arm pilot that the study team adapted from the CDC Diabetes Prevention Program[7] and American Heart Association Check, Change, Control programs applying informed by evidence-based strategies and stakeholder feedback.[8] Thus, participants will not be randomized and will all receive the intervention.[6] Our study will not be randomized due to: 1) this is a pilot program that has not been previously tested for feasibility and acceptability; 2) at the time of recruitment, we will have data over 5 years showing trends in ideal cardiovascular health metrics among AAMWI walk participants for baseline comparison (not a true study comparison group); and 3) concerns from the community of participants not receiving a potentially beneficial intervention (albeit, novel and not previously tested).

Intervention:
The intervention is a 24-week community-based lifestyle intervention to improve cardiovascular health among AA males involving two components: a CHW and peer team. Each participant will be assigned to a CHW and grouped into a team of 10 participants based on participant proximity to a central meeting location (i.e. Columbus Recreation and Parks centers and YMCAs). Each team's four health coaches will deliver content and coaching around the lifestyle intervention described below. The intervention will be applied evenly to all participants. Health coaches will implement the curriculum [Table 1] utilizing resources from the partnering organizations. Health coaches will meet with participant teams weekly for 75 minutes. Key health coach activities include delivering education, establishing and monitoring progress in achieving individual and team-based SMART (specific, measurable, achievable, relevant, and time-bound) wellness goals.

Table 1. Community-based lifestyle intervention curriculum

Weeks	Session topics	Partners	Study Measurements
1	• Introduction to Life Simple 7 and review of data on Life Simple 7 from the annual AAMWI walk (15 min)<br>• Overview of plan for and goals of the program (15 min)<br>• Purpose: Why did you join the program? What do I hope to achieve? How will healthy eating, physical activity, smoking cessation, blood pressure control, blood sugar control, blood pressure control, weight loss, help myself and others? (20 min)<br>• ***AHA Check, Change, Control, BP Check Principles (20 min)***<br>• How to keep track? Electronic vs. Paper (10 min)<br>• Physical activity assessment, instruction and plan development (10 min)	American Heart Association	**Complete Surveys online via redcap portal prior to Week 1, Labs and Vitals Week 1**
2	• **Life's Simple 7- Physical Activity (15 min)**<br>• DPP Session 5: Move Those Muscles (10 min)<br>• DPP Session 6: Being Active A Way of Life (20 min)<br>• Physical activity assessment, instruction and plan development (30 min)		Weight Check

**Table 1 continued. Community-based lifestyle intervention curriculum**			
Weeks	Session topics	Partners	Study Measurements
3	• **Life's Simple 7- Body Mass Index (15 min)**<br>• Goal Weight Target Assessment (7% weight loss if BMI > 25 kg/m^2 [See Session 1: DPP for Target Weights) (15 min)<br>• Physical activity assessment, instruction and plan development (45 min)		Weight Check
4	• **Life's Simple 7- Dietary Intake (DASH, American Heart Association and DPP dietary recommendations) (15 min)**<br>• DPP Session 2: Be a Fat, Calorie and Salt Detective (15 min)<br>• Physical activity (45 min)	OSU Nutrition Services<br>Philip Heit Center for Health and Wellness (Allan Sommer)<br>Franklin County Parks and Recreation Get Active Center	Weight Check
5	• **Life's Simple 7 - Blood Pressure (10 min)**<br>• ***AHA Check, Change, Control, BP Check (20 min)***<br>• Physical Activity (45 min)	American Heart Association	Weight, BP check
6	• **Life's Simple 7 - Cholesterol (10 min)**<br>• DPP Session 3 – Three Ways to Eat Less Fat and Fewer Calories (10 min)<br>• DPP Session 4 – Healthy Eating (10 min)<br>• Physical Activity (45 min)		Weight Check
7	• **Life's Simple 7 - Blood Sugar (20 min)**<br>• Review of how sessions 1-6 impact blood sugar (10 min)<br>• Physical Activity – (45 min)		Weight Check
8	• **Life's Simple 7 - Smoking (15 min)**<br>• Franklin County Smoking Prevention and Cessation (30 min)<br>• Physical Activity (45 min)	Franklin County Public Health	Weight Check
9	• **Cooking Demonstration – James Mobile Kitchen (45 min)**<br>• Physical Activity (45 min)		Weight, BP check
10	• DPP Session 7: Tip the Calorie Balance (15 min)<br>• DPP Session 8: Take Charge of What's Around You (15 min)<br>• Physical Activity (45 min)		Weight Check
11	• Stress Management and Mental Health Strategies & Resources (18 min)<br>• Intro to AAMWI Barbershop Talk: It's Ok to Talk (2 min)<br>• DPP Session 15: Stress Management (10 min)<br>• Physical Activity (45 min)	Columbus Area Integrated Services	Weight Check
12	• DPP Session 9: Problem Solving (15 min)<br>• DPP Session 10: Four Keys to Healthy Eating Out (15 min)<br>• Physical Activity (45 min)		**Complete Surveys online via redcap Labs and Vitals**
13	• DPP Session 11: Talk Back to Negative Thoughts (30 min)<br>• ***AHA Check, Change, Control, BP Check***<br>• Physical Activity (45 min)		Weight, BP check
14	• DPP Session 12: The Slippery Slope of Lifestyle Change (30 min)<br>• Physical Activity (45 min)		Weight Check
15	• Introduction to Clinical Trials (20 min)<br>• Community Scientist Academy program (10 min)<br>• Physical Activity (45 min)	OSU Center for Clinical and Translational Science	Weight Check
16	• DPP Session 13: Jump Start Your Physical Activity (30 min)<br>• Physical Activity (45 min)		Weight Check
17	• ***AHA Check, Change, Control, BP Check (30 min)***<br>• Physical Activity (45 min)		Weight, BP check
18	• DPP Session 14: Making Social Cues Work for You (30 min)<br>• Physical Activity (45 min)		Weight Check
19	• Money Matters: Financial Wellness Strategies (45 min)<br>• Physical Activity (30 min)	National Center for Urban Solutions	Weight Check
20	• Grocery Store Tour		Weight Check
**Table 1 continued. Community-based lifestyle intervention curriculum**			
Weeks	Session topics	Partners	Study Measurements

21	• Introduction to Global One Health Initiative (30 min)<br>• Physical Activity (45 min)	Global One Health Initiative	Weight, BP check
22	• DPP Session 16: Ways to Stay Motivated (30 min)<br>• Physical Activity (45 min)		Weight Check
23	• The importance of 7-8 hours of sleep (30 min)<br>• Physical Activity (45 min)		Weight Check
24	• Introduction to ResearchMatch (30 minutes)<br>• Physical Activity 45 min	OSU Center for Clinical and Translational Sciences	**Complete Surveys online via redcap Labs and Vitals**

Table 2. Project Timeline and Outcomes

	Year 1				Year 2			
**Milestone**	Q1	Q2	Q3	Q4	Q1	Q2	Q3	Q4
IRB approval	•							
Recruitment/enrollment	•	•						
CHW training	•	•						
Intervention			•	•				
Data analyses					•	•		
Dissemination/manuscripts							•	•

<u>Figure 3. Study Timeline</u>

<u>Data Measurements:</u>
The Reach, Efficacy, Adoption, Implementation, and Maintenance (RE-AIM) framework will guide evaluation of the intervention.[30–32] Effectiveness and maintenance: Life's Simple 7 measures (secondary outcomes) and HRQOL, Perceived Stress, Sleep Quality, Depressive Symptoms collected pre-mid-post-intervention. Adoption (acceptability): The participants will be asked to complete a survey of program satisfaction, including the content, length, and dose of the intervention. Implementation (treatment fidelity): We will follow the NIH's Behavioral Change Consortium treatment fidelity guidelines in study design, study staff training, treatment delivery, treatment receipt, and treatment enactment, to ensure treatment fidelity in the intervention.[33]

Data from participants will include self-report measures and survey data will be collected via paper or directly entered into Redcap. Biometric measurement will include blood pressure, cholesterol, glucose, and body mass which will be entered into Research Electronic Data Capture (REDCap) by health coaches. Data will be coded. After completion of data collection, the PI will retain the master list and subjects will be de-identified in data analyses and publications. Measure descriptions are included below:

<u>Outcome Measures:</u>

Primary Outcomes:
- Feasibility and Acceptability

Secondary Outcomes (Pre-Post):

- Prevalence of participants with a primary care provider
- Change in social and patient activation needs
- Change in cardiovascular health as per American Heart Association LS7 Metrics including blood pressure, cholesterol, glucose, smoking, body mass index, physical activity and dietary intake
- Prevalence of participants participating in OSU ResearchMatch.

i. We will measure feasibility via the study procedure logs (i.e., study enrollment and study contact completion).

We will assess protocol acceptability (satisfaction, usefulness, and plans and change of behavior to apply health promotion strategies). At the end of each program session, participants will complete a process evaluation via exit survey reviewed by stakeholders. We will use open-ended items to evaluate each participant's SMART goals and progress toward those goals. We will also inquire about what and how the participants like, dislike, and recommend intervention improvement. Specific questions will be: 1) What are your SMART Goals this week? 2) Do you have concerns about your ability to meet the goal(s) you set? If so, list your concern(s). 3) Have you changed your behavior as a result of the intervention" If so, how? 4) One a scale of 0 to 10 (with 0 being not at all satisfied and 10 being completely satisfied) how satisfied are you with this session? 5) On a scale of 0 to 10 (with 0 being not at all useful and 10 being most useful), how useful do you find this session? 6) What was the most useful part of this session? 7) Do you have any additional comments or suggestions?

ii. Validated Questionnaires will be assessed prior to intervention, 12 weeks (mid-point) and post intervention (24 weeks)

- Centers for Medicare and Medicaid Services Accountable Health Communities Health-Related Social Needs Screening Tool - The Centers for Medicare & Medicaid Services (CMS) Accountable Health Communities Model, tested by the Center for Medicare and Medicaid Innovation, addresses the critical gap between clinical care and community services in the current health care delivery system by testing whether systematically identifying and addressing the health-related social needs of Medicare and Medicaid beneficiaries impacts their total health care costs and improves health. The questionnaire includes 26 questions addressing living situation, food security, transportation, utilities, safety, financial strain, employment, family and community support, education, physical activity, substance abuse, mental health and disabilities.[9]

- Insignia Health Patient Activation Measure - The Patient Activation Measure (PAM) contains 13 items, segmenting individuals into one of four activation levels along an empirically derived 100-point scale. The four activation levels are 1) disengaged and overwhelmed, 2) becoming aware, but still struggling; 3) taking action and 4) maintaining behaviors and pushing further. Each level provides insight into an extensive array of health-related characteristics, including attitudes, motivators, and behaviors. Individuals in the lowest activation level do not yet understand the importance of their role in managing their own health, and

have significant knowledge gaps and limited self-management skills. Individuals in the highest activation level are proactive with their health, have developed strong self-management skills, and are resilient in times of stress or change. Each point increase in PAM score correlates to a 2% decrease in hospitalization and 2% increase in medication adherence. The questionnaire has acceptable reliability with α=0.80.[10–12]

- Diet History Questionnaire III - The Diet History Questionnaire (DHQ) is a freely available food frequency questionnaire (FFQ) for use with adults. The most recent version, DHQ III, can be used by researchers, clinicians, or educators to assess food and dietary supplement intakes. **It is available as a web-based tool only. Paper and pencil administration is not supported.** The nutrient and food group database for DHQ III is based on a compilation of national 24-hour dietary recall data from the National Health and Nutrition Examination Surveys (NHANES) conducted in 2007-08, 2009-10, 2011-12, and 2013-14 among those 19 years or older. Use in younger age groups is not appropriate given that the food list and portions are based on adult data. DHQ III is available as a web-based tool. DHQ III consists of 135 food and beverage line items and 26 dietary supplement questions. Some line items for foods and beverages have additional embedded questions that allow for final assignment to items in the nutrient and food group database leading to 263 foods/beverages listed in the database. https://epi.grants.cancer.gov/dhq3/ Prior research has shown that questionnaire is valid and reliable.[13–15]

- Center for Epidemiologic Studies Depression Scale (CES-D):[16,17] The CES-D scale is a brief self-report scale designed to measure self-reported symptoms associated with depression experienced in the past week. The items of the scale are symptoms associated with depression which have been used in previously validated longer scales. The CES-D has been shown to be a reliable measure for assessing the number, types, and duration of depressive symptoms across racial, gender, and age categories.[16,18,19] High internal consistency has been reported with Cronbach's alpha coefficients ranging from .85 to .90 across studies.[16]

- International Physical Activity Questionnaire -Short Form (IPAQ-SF): Physical activity will be measured using the International Physical Activity Questionnaire. The IPAQ is a 7-item questionnaire designed for adults ages 18-65, assessing time spent walking, in vigorous- and moderate- intensity activity and in sedentary activity in the past week. This measure has shown acceptable test-retest and criterion validity.[20]

- General Ethnic Discrimination Scale: The General Ethnic Discrimination Scale is an 18-item measure of perceived ethnic discrimination that can be used in health research with any ethnic group. The measure assesses both the frequency and the appraisal of discriminatory events in a manner that permits evaluation of the independent contribution of each to health and health behavior. The scale shows high reliability across racial/ethnic groups α~0.94.[21]

- Perceived Stress Scale - The Perceived Stress Scale (PSS) is a classic 10 question stress assessment instrument to measure the perception of stress. The questions in this scale ask about your feelings and thoughts during the last month. The scale is valid and reliable.[22,23]

- Pittsburgh Sleep Quality Index - The Pittsburgh Sleep Quality Index (PSQI) is an effective instrument used to measure the quality and patterns of sleep in adults. It differentiates "poor" from "good" sleep quality by measuring seven areas (components): subjective sleep quality, sleep latency, sleep duration, habitual sleep efficiency, sleep disturbances, use of sleeping medications, and daytime dysfunction over the last month. The measure consists of 19 individual items, creating 7 components that produce one global score, and takes 5–10 minutes to complete.[24] Cronbach's alphas range from 0.70 to 0.83, meeting the cut-point for a positive rating for within- and between-group comparisons. High correlations exist between PSQI with other measures of sleep quality (i.e., clinical diagnosis of insomnia, some variables of polysomnography and actigraphy, etc.). There is good construct validity.[25]

- RAND-SF 36 - The RAND Medical Outcomes Study (MOS) 36-Item Short-Form Health Survey (SF-36) [Version 1.0], consisting of 36 items included in long-form measures developed for the MOS. The SF-36 taps eight health concepts: physical functioning, bodily pain, role limitations due to physical health problems, role limitations due to personal or emotional problems, general mental health, social functioning, energy/fatigue, and general health perceptions. It also includes a single item that provides an indication of perceived change in health. It is a general measure of health-related quality of life. Reliability estimates for summary measures have exceeded 0.90.[26,27] Extensive support for the construct validity of the SF-36 summary scores has also been shown and thus this measure has been included in a numerous number of clinical trials and research studies across ethnicities and populations.[28]

iii. Blood pressure will be checked via an automated oscillometric sphygmomanometer (Omron 5 series), weight and height, blood cholesterol, blood glucose will be assessed for each participant prior to intervention, 12 weeks (mid-point) and post intervention (24 weeks).
iv. Blood cholesterol and blood glucose will be collected for each participant via finger stick pre-intervention, 12-weeks (midpoint) and post-intervention. The total amount of blood that will be collected for this entire research study (over 24 weeks) is less than 5 mL (less than 1 teaspoon).
v. Waist Circumference will be measured at the umbilicus-level waist circumference will be measured using a standard tape measure with 4 oz. tension.

Data Management

The database used for this study is REDCap, which is a secure web-based application for building and managing data. Data will be collected prospectively by study staff using the EMR and participant report as primary sources. It is designed specifically for clinical research and administered by the Center for Clinical and Translational Research. Permission for data access or entry will be granted or revoked at a level that is appropriate for each individual involved in the study. Following verification, data will be

locked. Data and data labels can be downloaded selectively (for interim progress reports) or in entirety (end of study) directly from Redcap in SAS or Excel format. The study team will record all baseline information and scheduled visits. Study team members will collect data from participants. Surveys/questionnaires will be administered using REDCap either via a confidential internet link or using a tablet/computer at/or prior to the first session.

Analysis and Internal Validity

Analytic Methods: The analytic strategy will use intent-to-treat (ITT) analysis. Preliminary analyses include descriptive statistics to check the data quality, examine variable distribution, and summarize sample characteristics. All data anomalies identified will be fully investigated and remedial strategies will be considered as appropriate.

Non-research related data previously collected at prior African American Male Wellness Walks will be used as part of this research project. We will analyze the data to evaluate longitudinal trends in the ideal cardiovascular health metrics for African American males participating in the walks.

Primary outcome: Feasibility and Acceptability
Descriptive statistics will be used to summarize quantitative data, including subject recruitment rate, retention rate, the degree of intervention adherence, and the average rating of the satisfaction of the intervention, including the content, length, and dose of the intervention. We will conduct content analyses on qualitative data obtained from verbatim transcripts of sessions and open-ended items of the battery. We will independently explore the data for patterns and significant statements that address intervention acceptability in NVivo 12 software. The research team will discuss, compare, and reduce codes iteratively until inter-coder agreement is met (kappa > 80%). Finally, we will use matrices to compare qualitative and quantitative acceptability data from the questionnaires. A mixed methods approach will lead to better understanding of how the intervention is perceived, along with which/how strategies affect outcomes in patient engagement and health-related outcomes. We expect that intervention is feasible, and AA men will perceive it as acceptable; however, findings may indicate further need to adapt the intervention (e.g., length of intervention).[34,35]

Secondary outcomes: 1) prevalence of participants with a primary care provider, change in social and patient activation needs; 2) Changes in cardiovascular health as per American Heart Association LS7 Metrics including blood pressure, cholesterol, glucose, smoking, body mass index, physical activity and dietary intake; and 3) prevalence of participants participating in OSU ResearchMatch. For each outcome, we will use mixed-effects linear modeling for repeated measures to (a) test the fixed effect of time, intervention group, and their interaction; (b) derive within-group and between-group contrast estimates on the change from baseline at each follow-up. Within-subject random effects will be included in the model to adjust for within-subject clustering from longitudinal follow-up. We will also adjust for potential confounders by including them as fixed-effects covariates in the model.

All statistical analyses will be performed using either SAS 9.4 or the most current version (SAS Institute, Inc., Cary, NC) or Stata 15 or the most current version (Stata Corporation, College Station, TX). Descriptive statistics (mean, median, standard deviation, proportions, etc.) of each of the collected variables will be estimated overall.

Sample Size and Power Analysis: This is a pilot study so sample size and power calculations were not performed. We will use data from this pilot study to solidify protocol parameters and estimate the degree of attrition that is considered in sample size and power calculation for a full-scale randomized controlled trial.

Missing Data: We expect missing data, for the post-intervention measures, where we expect a 10% attrition rate. We will carefully examine the pattern of the missing data and impute missing data via multiple imputation methods if missing completely at random (MCAR) mechanism is present. The mixed effects modeling approach described earlier allows for data missing at random (MAR). If the pattern of missing data indicates the potential missing not at random (MNAR), pattern-mixture modeling will be used. The robustness of findings under MCAR, MAR, and MNAR mechanisms will be evaluated.

Heterogeneity of Treatment Effect (HTE): To determine if there is HTE, we will extend our analysis models to include statistical interactions between participant factors and treatment and perform Wald tests of the interaction effect. Potential interaction factors include age, income, education level, neighborhood SES, and food insecurity. These analyses will be considered exploratory and thus no additional adjustment of p-values will be undertaken.

Confidentiality

Demographic, screening and survey data will be stored electronically behind the OSUWMC firewall in REDCap. Only the PI, co-investigators and the research assistants working on the project will have access to the data. All computers that will be used to store data will be password protected behind the OSUWMC. Electronic copies of forms will be stored on a secure dedicated server with appropriate firewalls. The system will use 128-bit encryption (SSL certificate) to transfer data between the machines. This technology is the same as that used for online e-commerce applications to protect consumer information such as name, address, and credit card details. Servers are scanned for viruses and systems are in-place to detect attempts at unauthorized entry. The data base server is backed up 3 times a day internally. Personal information will be stored confidentially for the minimum required amount of time after study completion.

Protection of Human Subjects

This human subjects research meets the definition of a clinical trial. We will not recruit from any vulnerable populations for this study.

I. Sources of Materials

Research Material obtained from living human subjects will include the following:

a. Finger stick samples: Cholesterol and Glucose, Pre-intervention, 12-weeks and Post-intervention

b. Data: Demographics and surveys completed during the course of the study and the anthropometric and clinical information collected (blood pressure, height, weight, etc.) pre-mid-post study.

Potential Risks:
1. Finger Stick cholesterol and glucose done at intervention site

Likely / Common

Mild

- Discomfort during finger prick
- Anxiety before and during finger prick
- Bleeding
- Bruising

The total amount of blood that will be collected for this entire research study (over 12 months) is approximately < 5 ml of blood. However, the tests are spread out over time, therefore, the risk of experiencing side effects is minimized.

Rare

- Infection at the site of blood drawing.

2. Injury during class i.e. burns or cuts – same procedure as #2.

Loss of confidentiality:
There is a potential risk that confidential information about the subject may be accidentally disclosed.

Confidentiality:
The research team will do everything we can to protect participant's privacy. Upon entry into the study, participants will be assigned a subject ID number. All of the study-related documents will be coded with this ID number so identities will be kept confidential. Study records will be stored in a locked area and only the study team will have access to them.

The research team will follow state and federal laws and may share information, only as necessary, with:
- Government representatives, (including the Office for Human Research Protections or the Food and Drug Administration) to complete federal or state responsibilities
- Hospital or University representatives to complete Hospital or University responsibilities
- Primary care physician if a medical condition that needs urgent attention is discovered
- The Research Subject Advocate at the CRU
- Regulatory agencies in other countries
- Other research laboratories working with us on this and other similar studies. When possible, the research team will make sure information cannot be linked to a specific individual.

Monitoring the Safety of Individual Participants

A. A research team member will be present during all testing procedures to monitor the safety of the individuals. A study team member will review any adverse events that may occur after or between visits. The primary investigator and co-investigators will monitor study progress on a weekly basis. Regular check-ins by the Principal Investigator

will be conducted with study team members to discuss any difficulties. All unanticipated problems will be reported to the IRB promptly. All events will be reviewed monthly by the PI and co-investigators. All serious adverse events (e.g. death or life-threatening adverse event) will be reported to the OSU Biomedical IRB verbally within 24 hours and written within 7 days of the event. Protocol deviations that may be expected to recur will also be reported immediately. Should there be a serious adverse event deemed related to the investigative treatment that increases the risk to participants, the study will be stopped, an investigation conducted, and findings generated before the study is resumed. These findings will be shared immediately with the IRB.

B. Description of anticipated adverse events (AE)
Definition of an AE: Any untoward medical occurrence in a subject in the intervention group, which could include a serious musculoskeletal injury requiring medical attention. All adverse events will be recorded in the study database including the following information:
1. the severity grade (mild, moderate, severe)
2. its relationship to the intervention
3. its duration (start and end dates or if continuing at final exam)
4. whether it constitutes a serious adverse event (SAE)

A SAE is any untoward medical occurrence that at any dose:
- results in death,
- is life-threatening,
- requires inpatient hospitalization or prolongation of existing hospitalization,
- results in persistent or significant disability/incapacity,
- is a congenital anomaly/birth defect,
- is otherwise a significant medical event.

Any SAE, irrespective of causality, occurring after the subject has provided informed consent and until four weeks after the subject has stopped study participation must be reported unless otherwise stated in the protocol. SAEs occurring after four weeks from ending study participation should only be reported if considered by the Investigator attributable to the exposure to the investigational drug(s) during the trial period. This includes the period in which the study protocol interferes with the standard medical treatment given to a subject, even if study treatment has not yet started (e.g. withdrawal of previous treatment during washout period, change in treatment to a fixed dose of concomitant medication).

C. Adverse event grading:

i. Identify the grading scale for AEs in this study:
AEs will be graded according to Mild, moderate, severe scale

ii. Identify the attribution scale to be used in this study:
The PI will determine the relationship of the AEs to the test intervention/agent(s)/device(s) as unrelated, possible related, probably related, or definitely related, using standard criteria for clinical trials**
**attribution in accordance with OSU ORRP-IRB criteria
(see http://www.orrp.ohiostate.edu/forms/osu_forms_table.html)

Definitely Related: An adverse event that has a timely relationship to the administration of the investigational intervention/agent/device and follows a known pattern of response for which no alternative cause is present.

Probably related: An adverse event that has a timely relationship to the administration of the investigational intervention/agent/device, however follows no known pattern of response but for which a potential alternative cause does not appear to exist.

Possibly Related: An adverse event that has a timely relationship to the administration of the investigational intervention/agent/device and follows a known pattern of response, but for which a potential alternative cause
may be present.

Unrelated: An adverse event for which there is evidence that it is definitely related to a cause other than the investigational intervention/agent/device; in general, no timely relationship to the administration of the intervention/agent/device exists, or if so, the event does not follow a pattern of response and an alternative
cause is present.

iii. Plan for reporting serious and/or unexpected adverse events:
Adverse event reporting will comply with University, as well as Federal guidelines [see 45CFR46.103(b)(5)(i); 21CFR312.32(c)(1)()], as appropriate. The incident reports that are submitted to the OSU ORRP-IRB.

In accordance with 45 CRF 312.64(b) any adverse effect that may reasonably be regarded as caused by the, or probably caused by, the intervention will be promptly reported by the investigator [researchers] to the OSU IRB. If the adverse effect is alarming, the adverse event will be reported immediately.

In all cases of serious and/or unexpected adverse events (SAE), the subsequent corresponding SAE report(s) will be sent to the IRB.

Each submitted AE Report will be in compliance with the appropriate HIPAA guidelines [e.g., not contain any personal identifiers of the study participant(s) for reports which will be disclosed, but will possess confidential participant identifiers (e.g., participant study identification number) that can be used by the investigator and study personnel to identify the participant (s)].

D. Plan for reporting adverse event (s):

i. OSU Office of Responsible Research Practices – Institutional Review Board,

ii. Study participant withdrawals/drop-outs:
Subjects who prematurely withdraw from the study due to an adverse event will be followed (e.g. telephone contact, and/or follow-up visits, etc.) until resolution of the event. Data and safety monitoring activities for this study will continue until all subjects have completed their participation and all subjects are beyond the time point at which study-related adverse events would likely present.

E. Plan for assuring data accuracy and protocol compliance:
The clinical research coordinator and/or investigator will be responsible for collecting and recording all relevant data for the protocol. As these results are collected and adverse events will be identified and reported to the principal investigator. Adverse events will be reported as described above. The principal investigator will determine the relationship of the event(s) to the intervention(s), procedure(s), and/or agent(s) of the protocol and decide the appropriate course of action for the study participant(s). Standard operating procedures for data collection and for conducting procedures will be developed. Data will be randomly audited (10% of subjects) by the study PI on an ongoing basis. Study staff will complete readily available training modules on the use of the REDCap database.

Nothing in the DSMP replaces an investigator's or study coordinator's responsibility for prompt and appropriate reporting of serious adverse events, protocol amendments, data collection procedures, etc. to the OSU ORRPIRB, sponsor(s), or other responsible parties. Any reporting required by the DSMP is in addition to these core compliance responsibilities.

Availability of Medical or Psychological Resources
If treatment is needed regarding participation in the research study, the participant will contact the PI/study team for needed care. The cost for this treatment will be billed to the participant or their medical or hospital insurance. The Ohio State University has no funds set aside for the payment of health care expenses for this study. If complications arise during study procedures, emergency facilities available through The Ohio State University Wexner Medical Center will be utilized.

III. Potential Benefits of the Proposed Research to the Subjects and Others

Benefits to participants potentially include improvements in LS7, HRQOL, stress, depressive symptoms, sleep, patient engagement and social determinants of health. Participants may also benefit from the knowledge that their participation would advance the understanding of the role of this intervention in improving the health of AA males. Participants will receive financial compensation. We expect this research to have a sustained and lasting impact on the health of AA males with poor or intermediate cardiovascular due to the new insights into the use of a community based participatory research intervention focused on cardiovascular health, as an approach to improve health outcomes for individuals with poor or intermediate cardiovascular health. The potential benefits and safeguards that are in place justify the above risks.

V. Importance of the Knowledge to be Gained

The results will further the understanding of this community-based participatory research intervention focused on cardiovascular health, as an approach to improve health outcomes for individuals with poor or intermediate cardiovascular health

VI. Bibliography

1. Lloyd-Jones DM, Hong Y, Labarthe D, Mozaffarian D, Appel LJ, Van Horn L, Greenlund K, Daniels S, Nichol G, Tomaselli GF, Arnett DK, Fonarow GC, Ho PM, Lauer MS, Masoudi FA, Robertson RM, Roger V, Schwamm LH, Sorlie P, Yancy CW, Rosamond WD, on behalf of the American Heart Association Strategic Planning Task Force and Statistics Committee. Defining and Setting National Goals for Cardiovascular Health Promotion and Disease Reduction: The American Heart Association's Strategic Impact Goal Through 2020 and Beyond. *Circulation*. 2010;121:586–613.

2. Dong C, Rundek T, Wright CB, Anwar Z, Elkind MSV, Sacco RL. Ideal cardiovascular health predicts lower risks of myocardial infarction, stroke, and vascular death across whites, blacks, and hispanics: the northern Manhattan study. *Circulation*. 2012;125:2975–2984.

3. Joseph JJ, Echouffo-Tcheugui JB, Carnethon MR, Bertoni AG, Shay CM, Ahmed HM, Blumenthal RS, Cushman M, Golden SH. The association of ideal cardiovascular health with incident type 2 diabetes mellitus: the Multi-Ethnic Study of Atherosclerosis. *Diabetologia*. 2016;59:1893–1903.

4. Rasmussen-Torvik LJ, Shay CM, Abramson JG, Friedrich CA, Nettleton JA, Prizment AE, Folsom AR. Ideal Cardiovascular Health Is Inversely Associated With Incident Cancer: The Atherosclerosis Risk in Communities Study. *Circulation*. 2013;127:1270–1275.

5. Givens M, Gennuso K, Jovaag A, Willems Van Dijk J, Johnson S. University of Wisconsin Population Health Institute. 2019 County Health Rankings. 2019;:16.

6. Melnyk BM, Morrison-Beedy D, eds. Intervention research: designing, conducting, analyzing, and funding. New York: Springer Pub; 2012: 1-473.

7. The Diabetes Prevention Program. Reduction in the Incidence of Type 2 Diabetes with Lifestyle Intervention or Metformin. *N Engl J Med*. 2002;346:393–403.

8. Bail J, Nolan TS, Vo JB, Gisiger-Camata S, Meneses K. Engaging an Urban African American Community to Deliver Cognitive Health Education to Breast Cancer Survivors. *J Cancer Educ*. 2018;33:870–874.

9. Centers for Medicare and Medicaid Services, Billioux A, Verlander K, Centers for Medicare and Medicaid Services, Anthony S, Centers for Medicare and Medicaid Services, Alley D, Centers for Medicare and Medicaid Services. Standardized Screening for Health-Related Social Needs in Clinical Settings: The Accountable Health Communities Screening Tool. *NAM Perspect*. 2017;7. doi:10.31478/201705b.

10. Hibbard JH, Stockard J, Mahoney ER, Tusler M. Development of the Patient Activation Measure (PAM): conceptualizing and measuring activation in patients and consumers. *Health Serv Res*. 2004;39:1005–1026.

11. Hibbard JH, Mahoney ER, Stock R, Tusler M. Do increases in patient activation result in improved self-management behaviors? *Health Serv Res*. 2007;42:1443–1463.

12. Ryvicker M, Feldman PH, Chiu Y-L, Gerber LM. The role of patient activation in improving blood pressure outcomes in Black patients receiving home care. *Med Care Res Rev MCRR*. 2013;70:636–652.

13. Thompson FE, Subar AF, Brown CC, Smith AF, Sharbaugh CO, Jobe JB, Mittl B, Gibson JT, Ziegler RG. Cognitive research enhances accuracy of food frequency questionnaire reports: results of an experimental validation study. *J Am Diet Assoc*. 2002;102:212–225.

14. Subar AF, Thompson FE, Kipnis V, Midthune D, Hurwitz P, McNutt S, McIntosh A, Rosenfeld S. Comparative Validation of the Block, Willett, and National Cancer Institute Food Frequency Questionnaires. *Am J Epidemiol*. 2001;154:1089–1099.

15. Kipnis V. Structure of Dietary Measurement Error: Results of the OPEN Biomarker Study. *Am J Epidemiol*. 2003;158:14–21.

16. Radloff LS. The CES-D Scale: A Self-Report Depression Scale for Research in the General Population. *Appl Psychol Meas*. 1977;1:385–401.

17. Radloff LS, Locke BZ. Center for Epidemiologic Studies Depression Scale (CES-D). In Handbook of Psychiatric Measures., 2nd ed. Washington, DC: American Psychiatric Publishing, Inc.; 2008.

18. Knight RG, Williams S, McGee R, Olaman S. Psychometric properties of the Centre for Epidemiologic Studies Depression Scale (CES-D) in a sample of women in middle life. *Behav Res Ther*. 1997;35:373–380.

19. Roberts RE, Vernon SW, Rhoades HM. Effects of language and ethnic status on reliability and validity of the Center for Epidemiologic Studies-Depression Scale with psychiatric patients. *J Nerv Ment Dis*. 1989;177:581–592.

20. Craig CL, Marshall AL, Sj??Str??M M, Bauman AE, Booth ML, Ainsworth BE, Pratt M, Ekelund U, Yngve A, Sallis JF, Oja P. International Physical Activity Questionnaire: 12-Country Reliability and Validity: *Med Sci Sports Exerc*. 2003;35:1381–1395.

21. Landrine H, Klonoff EA, Corral I, Fernandez S, Roesch S. Conceptualizing and Measuring Ethnic Discrimination in Health Research. *J Behav Med*. 2006;29:79–94.

22. Cohen S, Kamarck T, Mermelstein R. A global measure of perceived stress. *J Health Soc Behav*. 1983;24:385–396.

23. Cohen S, Kamarck T, Mermelstein R. Perceived stress scale. *Meas Stress Guide Health Soc Sci*. 1994.

24. Buysse DJ, Reynolds CF, Monk TH, Berman SR, Kupfer DJ. The Pittsburgh Sleep Quality Index: a new instrument for psychiatric practice and research. *Psychiatry Res*. 1989;28:193–213.

25. Mollayeva T, Thurairajah P, Burton K, Mollayeva S, Shapiro CM, Colantonio A. The Pittsburgh sleep quality index as a screening tool for sleep dysfunction in clinical and non-clinical samples: A systematic review and meta-analysis. *Sleep Med Rev*. 2016;25:52–73.

26. Hays RD, Sherbourne CD, Mazel RM. The rand 36-item health survey 1.0. *Health Econ*. 1993;2:217–227.

27. Hays RD, Morales LS. The RAND-36 measure of health-related quality of life. *Ann Med*. 2001;33:350–357.

28. Bize R, Johnson JA, Plotnikoff RC. Physical activity level and health-related quality of life in the general adult population: A systematic review. *Prev Med*. 2007;45:401–415.

29. Ricci-Cabello I, Ruiz-Perez I, Nevot-Cordero A, Rodriguez-Barranco M, Sordo L, Goncalves DC. Health Care Interventions to Improve the Quality of Diabetes Care in African Americans: A systematic review and meta-analysis. *Diabetes Care*. 2013;36:760–768.

30. Glasgow RE, Vogt TM, Boles SM. Evaluating the public health impact of health promotion interventions: the RE-AIM framework. *Am J Public Health*. 1999;89:1322–1327.

31. Glasgow RE, McKay HG, Piette JD, Reynolds KD. The RE-AIM framework for evaluating interventions: what can it tell us about approaches to chronic illness management? *Patient Educ Couns*. 2001;44:119–127.

32. Glasgow RE, Nelson CC, Strycker LA, King DK. Using RE-AIM Metrics to Evaluate Diabetes Self-Management Support Interventions. *Am J Prev Med*. 2006;30:67–73.

33. Bellg AJ, Borrelli B, Resnick B, Hecht J, Minicucci DS, Ory M, Ogedegbe G, Orwig D, Ernst D, Czajkowski S, Treatment Fidelity Workgroup of the NIH Behavior Change Consortium. Enhancing Treatment Fidelity in Health Behavior Change Studies: Best Practices and Recommendations From the NIH Behavior Change Consortium. *Health Psychol*. 2004;23:443–451.

34. Intervention research: Designing, conducting, analyzing, and funding: Springer Publishing Company; 2012.

35. Teddlie C, Tashakkori A. Foundations of mixed methods research. Thousand Oaks, CA: Sage; 2009.
